# Genetic diversity of *Aedes aegypti* populations from Kisumu and Busia counties, western Kenya, and their vector competence for chikungunya virus

**DOI:** 10.1371/journal.pone.0289191

**Published:** 2025-03-25

**Authors:** Victor O. Anyango, Solomon Langat, Francis Mulwa, James Mutisya, Hellen Koka, Collins Okoyo, Edith Chepkorir, Samson Konongoi, Anncarol Karanja, Glennah Kerubo, Rosemary Sang, Joel Lutomiah

**Affiliations:** 1 Department of Microbiology, School of Biochemistry, Microbiology, and Biotechnology, Kenyatta University, Nairobi, Kenya; 2 Division of Arboviruses and Viral Hemorrhagic Fevers Research, Center for Virus Research, Kenya Medical Research Institute, Nairobi, Kenya; 3 International Center of Insect Physiology and Ecology (ICIPE), Nairobi, Kenya; Beni Suef University Faculty of Veterinary Medicine, EGYPT

## Abstract

*Aedes aegypti* (*Ae. aegypti*) is the primary vector of several arboviruses, including dengue virus (DENV), chikungunya virus (CHIKV), yellow fever virus (YFV), and Zika virus (ZIKV). This vector is widespread globally in tropical and subtropical areas but also found in temperate areas. Kenya experienced its first chikungunya outbreak in Lamu County in 2004, followed by subsequent outbreaks in Mandera in 2016 and Mombasa in 2017. Despite the presence of *Ae. aegypti* in Kisumu and Busia counties, no outbreaks of chikungunya fever have been reported in these two western Kenya counties. To investigate this phenomenon, we collected *Ae. aegypti* mosquitoes from the county headquarter towns of Kisumu and Busia. The mosquitoes were reared under controlled laboratory conditions, and their genetic diversity assessed using COI gene sequences. Additionally, neutrality tests, including Tajima’s D and Fu’s FS, were subsequently performed to infer evolutionary dynamics. The mosquitoes were then evaluated for their ability to transmit CHIKV by challenging laboratory-reared F_1_ generations of field-collected mosquitoes with an infectious blood meal containing CHIKV. Genetic analysis revealed the presence of both *Ae. aegypti* subspecies, (*Ae. aegypti aegypti* [*Aaa*] and *Ae. aegypti formosus* [*Aaf*]) in the two western Kenya counties, with *Aaf* being dominant (19:8 for Kisumu samples and 25:6 for Busia samples). The populations exhibited high haplotype diversity (0.96011 in Kisumu and 0.93763 in Busia) and low nucleotide diversity (0.00913 in Kisumu and 0.00757 in Busia), indicating significant genetic polymorphism at the loci examined. Additionally, negative neutrality tests, including Tajima’s D (-1.87530 for Kisumu and -1.09547 for Busia) and Fu’s FS (-10.223 for Kisumu and -15.249 for Busia), coupled with a smooth mismatch distribution, suggest that recent evolutionary events may have significantly shaped the genetic structure of these populations. The assessment of vector competence of *Ae. aegypti* populations from Kisumu and Busia counties revealed their capacity to support CHIKV transmission. Specifically, we demonstrated infection, dissemination, and transmission rates of 55.2%, 85.5%, and 27.1% for Kisumu, and 57.8%, 71.8%, and 25% for Busia, respectively. However, statistical analysis indicated no significant difference in vector competence between the two populations. These findings underscore the uniform potential of *Ae. aegypti* mosquitoes from both Kisumu and Busia to facilitate the spread of CHIKV, highlighting the need for consistent surveillance and vector management strategies across these regions.

## Introduction

*Ae. aegypti* is the primary vector for chikungunya virus and is believed to have originated from Africa [1], where it was a single species inhabiting forests, breeding in tree holes, rock pools, and still water, and feeding on wild animals [2]. Later genetic analyses identified two morphologically, behaviorally, and genetically differentiated subspecies of *Ae. aegypti*: *Ae. aegypti aegypti* (*Aaa)* and *Ae. aegypti formosus* (*Aaf*) [2,3]. *Aaf* is darker in color, zoophilic and confined to forests; while *Aaa,* also referred to as the domestic subspecies is lighter, anthropophilic and breed in artificial habitats [2,4]. Research suggests that *Aaf* may have been brought to the New World aboard ships during the slave trade, where it evolved into *Aaa*, a more proficient carrier of arboviruses with a specialization for biting humans [3,5]. Consequently, *Aaa* subspecies is thought to have been introduced to the African continent through its seaports [2,6] and could have migrated to other parts of the region via the available modes of transport which was mainly the railway line. The frequent outbreaks of chikungunya and dengue fever, along Africa’s coastal areas lends credence to this hypothesis. Moreover, genetic analysis of *Ae. aegypti* mosquitoes has revealed that the sylvan form is more common in Africa, while the domestic form is prevalent in Europe, North and South America, and Asia [2,4].

*Ae. aegypti aegypti* has been shown to be more efficient in transmitting arboviruses than *Aaf,* due to its preference for human blood meal, and ability to feed multiple times during a single gonotrophic cycle [7–9]. Furthermore, Aubry *et al.* (2020) demonstrated that the *Aaf* [4] is less susceptible to ZIKV infection compared to the domestic form, *Aaa*. Additionally, Grard *et al.* (2014) demonstrated that the 2007 Zika virus disease outbreak in Gabon was driven by *Ae. albopictus* despite the presence of *Ae. aegypti* mosquitoes in the country, probably because this later species is predominantly *Aaf* subspecies [10]*.* Interestingly, the 2015 – 2016 Zika virus disease outbreaks in Brazil and the Caribbean were linked to the domestic form, *Aaa* [11]. These findings underscore the substantial heterogeneity in susceptibility and vector competence observed between the two *Ae. aegypti* subspecies highlighting the intricate dynamics of arbovirus transmission.

Chikungunya virus is a positive-sense RNA virus belonging to the family, *Togaviridae* and genus, *Alphavirus*. It was first identified in Tanzania in 1952 [12] and has since spread to more than 110 countries across different continents, including Africa, Asia, the Indian subcontinent, Europe, and the Americas [13–18]. CHIKV is primarily transmitted through bites of infected mosquitoes particularly the *Aedes* species and is often characterized by self-limiting symptoms that last 1 to 2 weeks and including febrile illnesses and severe arthritis. Instances of CHIKV have been reported in several regions of Kenya, including Lamu in 2004 and 2021-2022 [14,17], Mandera in 2016 [16] as well as in Mombasa in 2017 and 2018 [19]. Moreover, it is estimated that approximately 150 people in Kenya have died due to Chikungunya fever [14,20], making the virus an emerging significant public health concern.

Despite the occurrence of *Ae. aegypti* mosquitoes throughout Kenya, there have been no reported outbreaks of chikungunya fever in the country’s western region [21,22]. This suggests that the transmission and impact of CHIKV in this region have been inconsequential. However, the widespread distribution of the vector and detection of virus antibodies in this region indicate a potential risk of transmission [21,23]. Therefore, this study aimed to investigate the entomological factors that may explain the absence of reported CHIKV outbreaks in Western Kenya, while also examining the population structure of the vector. Specifically, we analyzed the genetic diversity and vector competence of *Ae. aegypti* populations from Kisumu and Busia to determine the genotypes present and to understand the mosquitoes’ ability to acquire and transmit the virus.

## Results

### Genetic diversity

Genetic analysis of *Ae. aegypti* mosquitoes collected from Kisumu (n = 27) and Busia (n = 31) counties revealed high levels of genetic diversity. In Kisumu, 18 unique haplotypes were identified, whereas Busia exhibited 23 unique haplotypes. This extensive genetic variation was underscored by high haplotype diversity (Hd >  0.5) observed in both populations, with values of 0.96011 in Kisumu and 0.93763 in Busia.

Despite the high haplotype diversity, nucleotide diversity (π) was low (π <  0.5) in both populations, with values of 0.00913 in Kisumu and 0.00757 in Busia. When the two populations were combined into a single dataset representing Western Kenya, a total of 58 sequences yielded 37 distinct haplotypes. The combined population exhibited a haplotype diversity of 0.96128 and a nucleotide diversity of 0.01136 (Table 1).

**Table 1:** Diversity table of the genetic diversity of *Ae. aegypti* mosquitoes from Kisumu and Busia counties (Western Kenya).

Sites	n	Hn	Hd	π	D	Fu’s Fs	r	R2
Kisumu	27	18	0.96011	0.00913	-1.87530	-10.223	0.0133	0.1207
Malaba	31	23	0.93763	0.00757	-1.09547	-15.249	0.0069	0.0762
Malaba & Kisumu (Western Kenya)	58	37	0.96128	0.01123	-2.16552	- 17.945	0.0105	0.0621

FST =  0.00575 |  Nm =  86.47.

n =  Number of sequences, Hn =  Number of haplotypes, Hd =  Haplotype diversity, **π** =  Nucleotide diversity, D =  Tajima’s D, Fu’s FS =  Fu’s selection test, r =  Raggedness statistic, R2 =  Ramos-Onsins and Rozas statistic.

Interestingly, the trend observed in the individual populations (high haplotype diversity coupled with low nucleotide diversity) was also evident at the regional level. This consistency suggests a shared demographic history or similar evolutionary pressures shaping the genetic structure of *Ae. aegypti* across western Kenya.

### Neutrality and mismatch distribution analysis

The population history of *Ae. aegypti* populations in the study regions was examined using several neutrality tests, including Tajima’s D, Fu’s FS, and mismatch distribution analysis. Tajima’s D values were negative in both Kisumu (-1.87530) and Busia (-1.09547), with a more pronounced negative value observed when the populations were combined (Western Kenya: -2.16552). This pattern suggests an excess of rare alleles and is consistent with population expansion. Fu’s FS analysis also revealed negative values in both Kisumu (-10.223) and Busia (-15.249), further supporting this hypothesis. Finally, mismatch analysis corroborated these findings, with low raggedness (r) and R² values characteristic of recently expanded populations (Kisumu: r =  0.0133, R² =  0.1207; Busia: r =  0.0069, R² =  0.0762), (Table 1). These results collectively suggest that recent evolutionary events, likely involving population expansion, have shaped the genetic structure of these populations.

We also conducted AMOVA to compare the genetic diversity of populations from Kisumu and Busia. The analysis revealed no significant genetic differentiation between the two populations, with only 0.62% of the total genetic variation observed between populations, and 99.38% within populations. However, it is important to acknowledge the limitations of this analysis, which included a relatively small sample size and the examination of only two populations.

### Phylogenetic analysis

**Fig 1:**
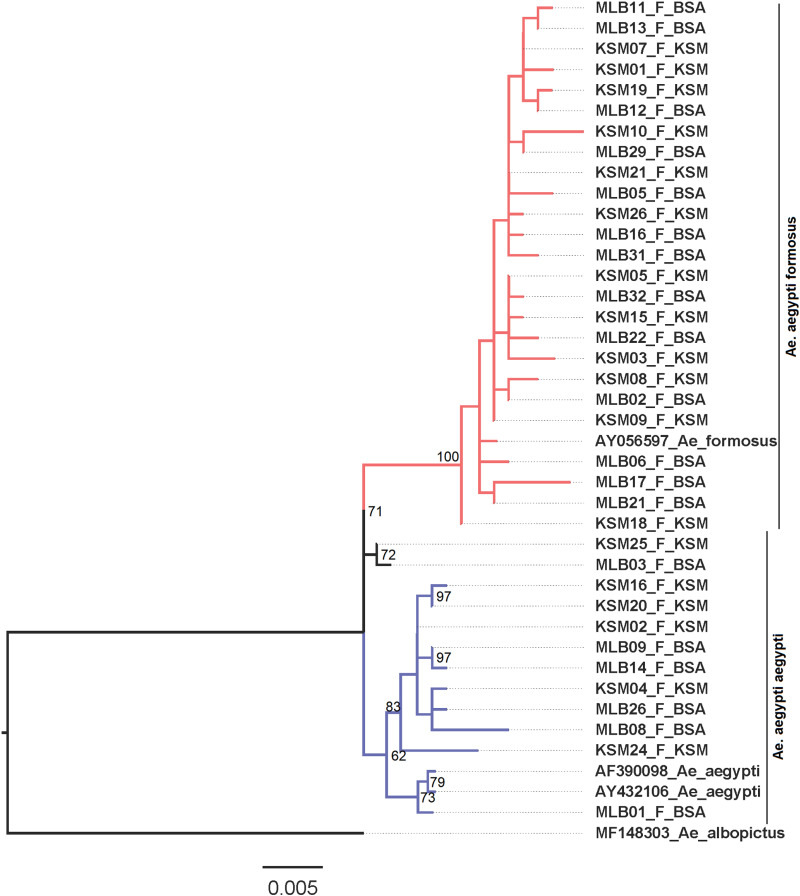
Maximum likelihood tree showing the genetic variability of *Ae. Aegypti* species collected from Kisumu and Busia Counties. The red and black colored branches represent haplotype sequences from the two sites clustered towards the *Aaf* genotype, while the blue colored branches represent those that clustered towards the *Aaa* genotype (the distinction has also been made by the two vertical lines on the far right of the diagram). The bootstrap supporting haplotypes that clustered towards the *Aaf* was 72% while that which supported haplotypes that clustered towards *Aaa* was 62%.

Phylogenetic analysis of the 18 and 23 haplotypes obtained from the Kisumu and Busia mosquito sequences showed the existence of the two *Ae. aegypti* sub species: *Aaa* and *Aaf* in both sites (Fig 1). *Aaf* were found to be more abundant in both sites compared to *Aaa*. Based on the phylogenetic analysis, there were no geographical clustering patterns of the sequences from either site, clustering was based on the sub species (*Aaa* and *Aaf*) as opposed to the study sites. Overall, there was high bootstrap support for the haplotype clustering which was above 50%.

### Vector competence of *Ae. aegypti* mosquitoes from Kisumu and Busia counties

A total of 260 mosquitoes from the two study sites were challenged with Log 10^6.79^ PFU/ml of CHIKV. Overall, 56.5% of the tested mosquitoes were susceptible to CHIKV, of which 78.2% disseminated the virus. Among those with disseminated infections, 26.1% exhibited transmission capabilities, as indicated by the presence of CHIKV in their saliva.

One hundred and twenty-five mosquitoes from Kisumu (1^st^ replicate [n =  42], 2^nd^ replicate [n =  42], 3^rd^ replicate [n = 41]) were challenged with CHIKV infectious blood meal. Of these, 55.2% became infected, of which 85.5% disseminated the virus. Notably, only 27.1% of those that successfully disseminated the virus demonstrated transmission capabilities. Similarly, a total of 135 (1^st^ replicate [n =  45], 2^nd^ replicate [n =  45], 3^rd^ replicate [n =  45]) mosquitoes from Busia were challenged with CHIKV infectious blood meal. Of these, 57.8% were infected, with 71.8% of the infected demonstrated disseminated infection, and 25.0% of mosquitoes with disseminated infections exhibited transmission capabilities.

Cytopathic effect (CPE) of Vero cell lines inoculated with mosquito body, leg and saliva samples was used as a measure of infection, dissemination, and transmission abilities of the test mosquitoes. It is important to note that CPEs were observed within 2 to 3 days post-inoculation for all test mosquitoes challenged with CHIKV. Interestingly, the proportional test of differences showed no significant differences between infection, dissemination, and transmission rates of mosquito populations from Kisumu and Busia (Table 2).

**Table 2:** Vector competence of *Ae. aegypti* mosquitoes from Kisumu and Busia counties for CHIKV.

Site	The proportion of mosquitoes infected with CHIKV. % (95%CI); n = 260		
	Body (Infection)	Leg (Dissemination)	Saliva (Transmission)
Overall	56.5 (50.8-62.9); n = 147	78.2 (71.8-85.2); n = 115	26.1 (19.2-35.5); n = 30
Busia (n = 135)	57.8 (50.0-66.7); n = 78	71.8 (62.5- 82.5); n = 56	25.0 (15.9- 39.4); n = 14
Kisumu (n = 125)	55.2 (47.1- 64.6); n = 69	85.5 (77.5-94.2); n = 59	27.1 (17.8- 41.2); n = 16
Proportion test of the difference (diff,, p value	Diff = 0.026, p = 0.751	Diff = -0.137, p = 0.072	Diff = -0.021, p = 0.896

N =  No. of mosquitoes, CI =  confidence interval, p - probability value, diff - difference between two values or groups being compared.

The study further compared rates of infection, dissemination, and transmission based on the virus incubation period in the sample mosquitoes. For Kisumu, approximately 44.2% of the mosquitoes processed after 5 days of incubation were infected with CHIKV. Of those infected, 78.9% disseminated the virus, 33.3% of which showed transmission capabilities. At 10-day post infection (dpi), approximately 60.9% of the processed mosquitoes were infected with CHIKV. Of those infected, 89.3% disseminated the virus, of which 40%tion showed transmission capabilities. Similarly, at 14-dpi, approximately 61.1% of the processed mosquitoes were infected with CHIKV, and 86.4% of those infected disseminated the virus. However, only 5.3% of those that disseminated the virus showed transmission capabilities by the presence of CHIKV in their saliva. (Table 3).

**Table 3:** Vector competence of *Ae aegypti* mosquitoes from Kisumu and Busia counties for CHIKV based on incubation periods.

Day	Kisumu					Busia				
	Day 5	Day 10	Day 14	Proportion test of difference at day 10	Proportion test of difference at day 14	Day 5	Day 10	Day 14	Proportion test of difference at day 10	Proportion test of difference at day 14
Body	44.2 (31.6-61.8); n = 19/43	60.9 (48.3-76.7); n = 28/46	61.1 (47.1-79.3); n = 22/36	Diff = 0.167, p = 0.259	Diff = 0.169, p = 0.280	59.6 (47.1-75.4); n = 28/47	48.9 (36.3-65.9); n = 22/45	65.1 (52.3-81.0); n = 28/43	Diff = 0.107, p = 0.450	Diff = -0.055, p = 0.671
Leg	78.9 (62.6-99.6); n = 15/19	89.3 (78.5-101.5); n = 25/28	86.4 (73.2-102); n = 19/22	Diff = 0.104, p = 0.367	Diff = 0.075, p = 0.562	62.1 (46.7-82.5); n = 18/27	81.8 (67.2-99.6); n = 18/22	74.1 (59.3-92.6); n = 20/27	Diff = 0.197, p = 0.199	Diff = -0.12, p = 0.427
Saliva	33.3 (16.3-68.2); n = 5/15	40.0 (24.7-64.6); n = 10/25	5.3 (7.8-35.5); n = 1/19	Diff = 0.067 p = 0.801	Diff = 0.279, p = 0.572	44.4 (26.5-74.5); n = 8/18	16.7 (59.3-46.8); n = 3/18	15.0 (52.8-42.6); n = 3/20	Diff = 0.277, p = 0.396	Diff = 0.294, p = 0.367

For mosquito populations from Busia County, 59.6% of those challenged with CHIKV were infected at 5-dpi, 62.1% of which disseminated the virus, and 44.4% of those that disseminated the virus showed transmission capabilities. The infection rate decreased from 59.6% at 5-dpi to 48.9% at 10-dpi, while the dissemination rate increased from 62.1% to 81.8%. However, the proportion of mosquitoes that showed transmission capabilities decreased significantly to 16.7% given the high number of mosquitoes that disseminated the virus. At 14-dpi, the infection, dissemination, and transmission rates were 65.1%, 74.1% and 15% respectively (Table 3).

The results of the plaque assay performed on positive mosquito bodies, legs and saliva from the Kisumu and Busia samples confirmed the presence of live viral particles, with mean titers of 10^5.230^ and 10^4.146^ for Kisumu bodies and legs as well as 10^5.38^ and 10^3.61^ for Busia bodies and leg respectively. These findings corroborate the cell culture results above (Table 4).

**Table 4:** Mean CHIKV titer in mosquito bodies and legs following exposure to infectious blood meal.

Site	Mean body titers (PFU/ML)	Mean Leg titers (PFU/ML)
Kisumu	10^5.230^	10^4.146^
Busia	10^5.380^	10^3.361^

## Discussion

Our study observed high haplotype diversity in Kisumu (0.96011 and Busia (0.93763 in) and low nucleotide diversity in Kisumu (0.00913) and Busia (0.00757) within *Ae. aegypti* populations from both counties. However, combining the two populations resulted in a modest increase in nucleotide diversity (0.01136) while maintaining high haplotype diversity (0.96128), reflecting a broader genetic variation at the nucleotide level across the western Kenya region.

In their classification of genetic diversity of populations based on mtDNA haplotype and nucleotide diversity, Grant and Bowen (1998) considered values >  0.5 as high and those <  0.5 as low [24], resulting in four key categories; (i) low *Hd* and low π (ii) high *Hd* and low π (iii) low *Hd* and high *π* (iv) high *Hd* and high π [25]. Our findings fall within the high *Hd* and low π category and align with several studies, where haplotype and nucleotide diversity values fall within the same range and have been similarly categorized [25–28]. The observed high haplotype diversity alongside low nucleotide diversity indicates a high degree of genetic variation within the two populations, primarily driven by the presence of numerous haplotypes with subtle differences at the nucleotide level.

The findings suggest a possible scenario in which the populations experienced bottleneck events that drastically reduced their sizes, followed by a significant increase in numbers. During this expansion, the retention of new mutations may have been enhanced, leading to high variability in haplotypes even though overall nucleotide variation remains low [24,25]. Another possible explanation is gene flow from genetically similar populations which may have introduced new haplotypes into the populations without a significant increase in nucleotide diversity [25,26,28].

To better understand the factors influencing the genetic diversity of *Ae. aegypti* in western Kenya, we performed several neutrality tests. Our analyses, including Tajima’s D, Fu’s FS, and mismatch distribution, consistently suggest that recent evolutionary events may have shaped the genetic structure of these populations. The negative Tajima’s D (Kisumu: -1.87530; Busia: -1.09547) and Fu’s FS values (Kisumu: -10.223; Busia: -15.249) tests observed in the study populations suggest an excess of rare alleles, a characteristic signature of various evolutionary processes, including population expansion, gene flow, selective sweeps, and bottlenecks [25,29]. However, mismatch analysis provides crucial additional information. The observed low raggedness statistics (r) and R² values coupled with the high haplotype and low nucleotide diversity across both populations point towards a recent and rapid population expansion in the study sites (western Kenya) [30].

Our analysis of phylogenetic relationship between the study samples revealed the existence of both *Aaa* and *Aaf* sub-species in the two cities, with *Aaf* being dominant (19:8 for *Aaf: Aaa* in Kisumu and 25:6 for *Aaf: Aaa* in Busia). These findings are in tandem with a previous study by Futami *et al*. (2020) which assessed the geographical distribution of *Aaa* and *Aaf* in Kenya [21]. The study found that *Aaa* was more prevalent in the coastal region, with decreasing abundance as distance from the sea increased towards Western Kenya where higher *Aaf* abundance was recorded. The presence of *Ae. aegypti* in diverse ecological settings within western Kenya, including urban and rural areas in and around Kisumu, has also been confirmed by other field studies [31–33]. Collectively, these results suggest that the distribution and abundance of *Aaa* and *Aaf* sub-species in Western Kenya may have implications for disease transmission in the region, since *Aaa* has been documented as an efficient vector for arboviruses due its anthropophilic behavior [2] and higher vector competence [34] than the zoophilic *Aaf*. Therefore, our findings highlight the importance of continued surveillance and monitoring of mosquito population dynamics and virus circulation in the region for early detection, outbreak prevention and mosquito control.

Our research further investigated the ability of *Ae. aegypti* mosquitoes from both study locations to transmit CHIKV. In general, our findings demonstrated that, *Ae. aegypti* mosquitoes from both locations are competent vectors for CHIKV. Despite the overall susceptibility of *Ae. aegypti* mosquitoes to CHIKV infection observed in both study sites, a significant proportion of infected mosquitoes successfully disseminated the virus. However, a notable observation was that only a small subset of mosquitoes with disseminated infection were ultimately capable of transmitting the virus. The possible role of midgut infection (MIB) and escape (MEB) barriers as well as salivary gland infection (SGIB) and escape (SGEB) barriers in explaining this phenomenon cannot be overlooked [35]. The average infection rate observed (56.5%) in this study may be an indication of a well-established and strong MIB in mosquitoes that failed to establish CHIKV infection. These barriers, created by the midgut epithelial lining, peritrophic matrix and midgut microbiota have been associated with reduction in vector viremia as they limit/prevent the establishment of pathogens within the mosquito’s body [35,36]. Moreover, of all the susceptible mosquitoes, 56.5%, 78.2% disseminated the virus, an indication of a possible weakened MIB and MEB among this population. Conversely, only 26.1% of the mosquitoes that disseminated the virus demonstrated transmission capabilities, suggesting an overall strong SGIB and SGEB among the populations. Salivary gland infection and escape barriers are responsible for preventing the establishment of pathogen in mosquitos’ salivary glands as well as the shedding of the pathogen into the mosquito saliva for transmission [36]. Therefore, these data underscore the need for additional studies on the role of barriers, as critical determinants of vector competence in mosquito populations. There is also increasing evidence that mosquito microbiota have a protective effect against viral infections [37], therefore the impact of mosquito microbiota on vector competence for CHIKV within these populations warrants further exploration.

It is important to note that the forced salivation procedure used in this study has been documented as a less efficient method for determining mosquito transmission abilities, given its propensity to underestimate virus transmission [38]. The presence of virus particles in mosquito legs is a more reliable predictor of its transmission capabilities than forced salivation methods [38]. Therefore, the findings of this study may underestimate the true extent of virus transmission, as transmission levels in natural settings with vertebrate hosts present could be higher compared to laboratory settings that utilize artificial capillaries.

A study by Agha *et al.,* (2017) demonstrated that, *Ae. aegypti* populations from Kisumu were competent to CHIKV further supporting the findings of this study [31]. Our study also demonstrated that CHIKV-infected mosquitoes could transmit the virus as early as 5-dpi, an indication of the study mosquito’s ability to efficiently transmit the virus within a short period of time, posing a substantial risk for the rapid spread of the virus within a human population. These findings align with previous studies that also observed a shorter incubation period of CHIKV in mosquito vectors [31,39].

It is important to note that literature has documented *Aaa* as the more efficient vector of some of the most common arboviruses including YFV, ZIKV, DENV and CHIKV. This has been mainly due to its anthropophilic nature and close association with human habitats [7–9]. on the other hand, *Aaf* primarily found in forested areas have been viewed as less efficient vector for these viruses and this may not necessarily mean that they are not capable of transmitting the virus but their interaction with humans may be less pronounced than *Aaa* [2]. In this study, we identified more *Aaf* in the two study sites than *Aaa* and also found that our study populations were competent to transmit CHKV. This implies that *Aaf* may be a competent vector for CHIKV in western Kenya, challenging its traditional view of a poor vector. However, the role of local environmental factors, genetic variability and ecological adaptation in facilitating this phenomenon cannot be overlooked and may need further investigation to ascertain its potential as a vector of arboviruses in specific ecological contexts.

In conclusion, our study provides key insights into the genetic diversity and vector competence of *Ae. aegypti* in Kisumu and Busia (western Kenya). We confirm the presence of both *Aaa* and *Aaf* subspecies in the region and demonstrate compelling evidence for a recent and rapid population expansion, characterized by high haplotype diversity, low nucleotide diversity, negative Tajima’s D and Fu’s FS values, and a smooth mismatch distribution. This expansion, likely influenced by ecological factors such as increased breeding sites due to urbanization or altered rainfall, and potentially by recent colonization or changes in mortality rates, has shaped the genetic structure of these populations.

We also demonstrate that, *Ae. aegypti* populations from both Kisumu and Busia are competent for CHIKV. This finding, combined with the evidence of population expansion, has significant implications for arbovirus transmission. The increased mosquito population size directly elevates the risk of outbreaks of diseases like dengue, Zika, chikungunya, and yellow fever in western Kenya. Furthermore, the existing high haplotype diversity represents a substantial reservoir of genetic variation that could facilitate future adaptation including the evolution of insecticide resistance. The similar genetic patterns observed in Kisumu and Busia, and the stronger signal in the combined western Kenya samples, suggest regional connectivity, underscoring the need for coordinated vector control strategies.

To further elucidate the complex relationship between genetic diversity, vector competence, and arbovirus transmission, particularly at the subspecies level, we recommend future research employing higher-resolution molecular markers such as Whole Genome Sequencing (WGS) or Single Nucleotide Polymorphism (SNPs) analysis across a wider geographic range in Kenya as this will provide a more comprehensive understanding of the spatial distribution and transmission dynamics of *Ae. aegypti*.

## Materials and methods

### Ethical statement

This study was approved by Kenya Medical Research Institute (KEMRI)’s Scientific Ethics Review Unit (SERU) under approval number, SERU 3433. Due to the nature of the study, informed consent was not required.

### Study sites

**Fig 2:**
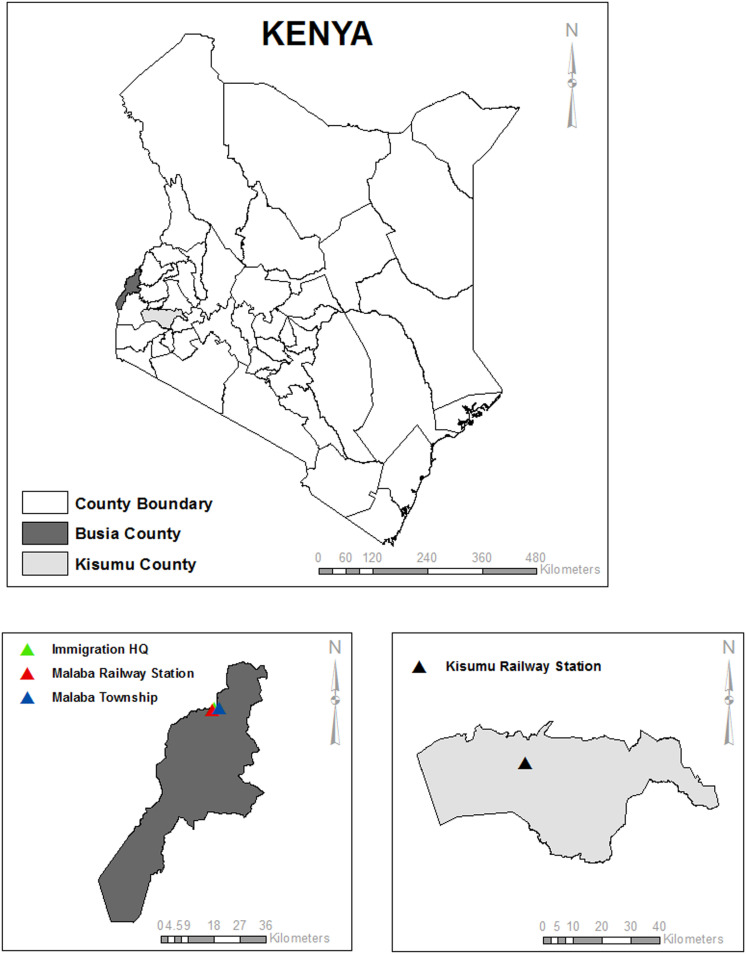
Map of Kenya showing the two study sites (Kisumu and Busia counties). Samples were collected in, Malaba railway station (red), and Malaba immigration center (green) and Malaba township (blue) for Busia mosquitoes while in Kisumu samples were collected in different locations within the old railway station just next to Lake Victoria (black).

The study was conducted in two counties, Kisumu and Busia, in western Kenya (Fig 2). Kisumu and Busia have a population of approximately 1,155,574 and 893,681 respectively according to the Kenyan population census of 2019 [40]. The climate comprises a warm and humid tropical climate with temperatures ranging from 22°C to 30°C for Kisumu and 18°C to 34°C for Busia throughout the year making the two counties conducive habitats for mosquitoes.

### Mosquito collection and rearing

From June to September 2021, *Ae. aegypti* mosquitoes were collected in Kisumu and Busia County headquarters as eggs, larvae, pupae, and adults from areas surrounding the old railway stations. Adult mosquitoes were collected using carbon baited BG sentinel traps during the day as well as at night. For the eggs collection oviposition cups (ovitraps) lined with oviposition papers and half-filled with water were used [41]. The ovitraps were placed under trees, bushes, residential and commercial areas, and old vacated railway buildings, for three days to allow mosquitoes to lay eggs. In addition, the immature stages of the mosquito: larvae and pupae were collected from various natural and artificial habitats such as tree holes, old tires, concrete holes, abandoned cans, and abandoned water tanks using scoopers and teat pipettes, and transferred into whirl-pak bags. Both the adult and immature mosquitoes were transported to the biosafety level II insectary at KEMRI in Nairobi, Kenya, for further analysis.

The identified F_0_ *Ae. aegypti* were reared under controlled insectary conditions (28 ±  1°C, 12:12 hour light-dark cycle, and 75 ±  5% relative humidity [RH]) with continuous access to 10% glucose and later used genetic analysis [42]. Field-collected eggs were hatched in a 24 ×  34 ×  9 cm tray half filled with dechlorinated water, larvae were maintained using standard Tetramin (Tetra) fish food and monitored daily for the presence of pupa. Pupae were picked from the tray and transferred to a 200ml beaker containing clean, dechlorinated water, which was then placed in an insect cage (BugDorm) measuring 30 x 30 x 30 cm and allowed to emerge into adults [42]. Approximately 600 pupae were placed in single cages [42]. Emerging adults were identified under a dissecting microscope and *Ae. aegypti* species isolated, blood-fed, and F_1_ eggs collected for subsequent vector competence assays.

### Assessment of genetic diversity *Ae. aegypti*

#### DNA extraction.

Approximately 32 field collected female mosquitoes (F_0_) were selected from each county (Kisumu and Busia) by sampling from various collection sites and collection methods. Individual mosquito legs were then isolated using sterile forceps and placed in a sterile RNase/DNase-free Eppendorf tube containing 3 pieces of 2.0 mm Zicornia beads. 200ml of Phosphate buffered saline was then added into each Eppendorf tube and the sample homogenized in an Omni Bead Rapture 24 (OMNI International) set to run at the speed of 2.10 mm for 20 seconds. *Quick-*DNA*™* Miniprep Kit (ZYMO RSEARCH) [43] was then used to extract DNA from the homogenates following the manufacturers directives.

#### Polymerase chain reaction.

Polymerase Chain Reaction (PCR) was used to amplify the target mtDNA (COI) in mosquito samples using the universal reverse primer HCO2198 and forward primer LCO1490 [44]. The PCR master mix was prepared in a Biosafety level II molecular laboratory and contained 12.5 µl Amplitaq solution, 1 µl forward primer, 1 µl reverse primer, and 8.5 µl sterile nuclease free water. Next, 23µl of the master mix was transferred into well-labelled RNase-free, 0.2 ml (8-strip format, Invitrogen) PCR tubes and 2 µl of sample DNA added into each tube. The tubes were tightly sealed, vortexed, and placed in a Thermocycler set to run 40 reaction cycles of 92°C for the 30s, 43–52°C for 30s, and 72°C for 60s [29]. The amplicons were visualized in a 2% agarose gel with an expected band size of approximately 650 bp, stained with ethidium bromide and sent to Macrogen Europe for sequencing [45].

### Determination of vector competence of *Ae. aegypti* to CHIKV

#### Virus amplification and quantification.

Chikungunya virus strain isolated during the 2018 outbreak in Mombasa, Kenya was used in the study [19]. The virus was obtained from KEMRI’s, Sample Management and Receiving Facility (SMRF) and amplified by inoculating 200µl into T-25 cell culture flask (Corning Incorporated, USA) containing 70% to 80% confluent Vero cells (ATTC CCL-81). The inoculation was incubated for 1 hour at 37°C and overlayed with 5ml of Maintenance medium containing 1X Minimum essential media (MEM), (GIBCO), supplemented with earl salt, 2% of 200Mm L-glutamine (Sigma-Aldrich), and 2% penicillin-streptomycin solution containing 10,000unit/ml penicillin and 10000ug/ml streptomycin (GIBCO) and 2% Fetal Bovine Serum (FBS) (GIBCO, UK). The inoculated flasks were further incubated in a 5% CO_2_ incubator set at 37°C and observed daily for CPE (22). At 80% CPE, the virus was harvested. Briefly, the virus-infected cells were frozen at -80°C, thawed, and centrifuged at 12,000 rpm for 10 minutes. Next, the supernatant, containing the virus particles, was collected and transferred to multiple 1.5 µl cryovials and the viral titer quantified using plaque assay as described in Agha *et al*. (2017) [31]. The ready-to-use virus aliquots were then stored at -80°C.

#### Mosquito exposure to infectious blood meals.

To ensure reproducibility and robustness of the results and minimize bias, all mosquito infection experiments were conducted in triplicates, with each experiment involving infection of separate group of mosquitoes following the same protocol and conditions. Briefly, four-day-old female *Ae. aegypti* mosquitoes deprived of sucrose solution for 24 hours were fed with an infectious blood meal consisting of one volume of defibrinated sheep blood and one volume of CHIKV. The mosquitoes were allowed to feed on the infectious blood meal for a duration of 45 minutes using an artificial membrane feeding system (Hemotek), adjusted to maintain the temperature of the blood-virus mixture at 37°C throughout the feeding period. An aliquot of the artificial blood meal was pipetted into a 1.5 ml sterile Eppendorf tube before the blood feeding process, and promptly stored at -80°C [42] for subsequent titration using plaque assay as described in Agha *et al* 2017 [31,46]. At the end of the 45-minute feeding period, fully engorged mosquitoes were transferred to a clean BugDorm-1 insect rearing cage and maintained under controlled laboratory conditions (28 ±  1°C, 12:12 hour light-dark cycle, and 75 ±  5% RH) with constant access to 10% sucrose solution [42].

#### Susceptibility and dissemination assays.

A sample of the study test mosquitoes that were incubated after exposure to infectious blood meals were tested at 5-, 10- and 14-dpi and inactivated by placing them in a -20 freezer for 20 seconds. Individual mosquitoes were then decapitated, by removal and transfer of their legs into a clean 1.5 ml Eppendorf tube containing 500µl of homogenization media (HM). Mosquito bodies were then immobilized on a sticky surface, and their proboscises individually inserted into a capillary tube containing HM to stimulate salivation. The mosquitoes were left immobilized for 30 minutes, after which the capillary tubes were removed, and their content dispensed into a well-labeled 1.5ml Eppendorf tube containing 200 µl of HM. The mosquito bodies were then picked using sterile forceps and placed in different tubes each also containing 500ml of HM. The Eppendorf tubes containing mosquito legs, body, and saliva samples were stored at -80°C awaiting cell culture assay.

#### Cell culture and inoculation.

Individual mosquito bodies stored at -80°C above were thawed on ice and homogenized using 4.5 mm copper beads in an Omni Bead Rapture 24 (OMNI International) set to run at the speed of 2.10 m/s for 20 seconds. Following the homogenization step, the homogenates were subjected to centrifugation at 12000 rpm for 10 minutes and the resulting supernatant carefully isolated and transferred into a clean cryogenic vial, ensuring the exclusion of any solid particles. Subsequently, the supernatants were used to inoculate 24-well plates containing ATTC CCL-81 Vero cells that had reached a confluence of 70-80%. For each well, 50μl of the supernatant was added and plates incubated in a 5% CO_2_ incubator for 1 hour with a 15-minute rocking interval to allow for adsorption. Next, 1ml of cell maintenance media (1X MEM [GIBCO], with supplements as described above under “virus amplification and quantification” was added and the plates returned to the 5% CO_2_ incubator [39]. The cells were observed daily under a microscope for the development of CPE, as an indicator of infection. Wells that showed 80% CPE were considered positive of the virus used to infect the mosquito, and the mosquitoes used to inoculate the cells deemed susceptible to CHIKV. Next the legs whose bodies showed CPEs were homogenized, and the above procedure repeated. Mosquito legs that showed 80% CPE were considered to have disseminated the CHIKV [31,39]. The same process except for the homogenization step was followed for saliva samples of mosquitoes whose legs showed CPE and mosquitoes whose saliva showed CPE were considered to have transmission capabilities.

### Data management and analysis

All data were meticulously collected following the aforementioned procedures. The genetic diversity data were systematically organized and stored in a well-labeled folder to ensure easy retrieval and traceability. Similarly, all data from the vector competence triplicate experiments were populated in one Excel file and securely stored in a separate folder. These datasets were then subjected to rigorous analysis as outlined below, and the outcomes are comprehensively presented in this paper.

#### Genetic diversity data.

The raw sequences were initially edited and converted into FASTA format sequences using Chromas v2.6.6 (Technelysium). The Basic Local Alignment Search Tool (BLAST) was then employed to compare the trimmed sequences against the GenBank database, with only sequences showing 98-100% similarity to *Ae. aegypti* accepted for further analysis. This similarity threshold was set to confidently confirm species identity and exclude potential contaminants.

Following identification, the sequences were aligned using the Muscle algorithm integrated in MEGA7, ensuring accurate sequence alignment across all samples. Aligned sequences were then categorized by study populations (Kisumu and Busia) to facilitate population-specific analyses.

To assess genetic diversity, we analyzed nucleotide variation within each population using DnaSP v6.12. This analysis provided key genetic diversity metrics, including the number of haplotypes (H), haplotype diversity (Hd), and nucleotide diversity (π) for each population. These metrics enabled a comprehensive evaluation of genetic diversity within the *Ae. aegypti* populations from Kisumu and Busia. Additionally, we conducted neutrality tests, including Tajima’s D and Fu’s FS, within DnaSP to assess the potential evolutionary forces shaping these populations.

Pairwise differences and population structures were evaluated by analysis of molecular variance (AMOVA) in Arlequin 3.5.2.2, and significance was evaluated based on 10,000 permutations. The gene flow between the two localities was estimated from pairwise FST and Nm values using the DnaSP software program. For phylogenetic analysis, sequences belonging to *Ae. aegypti aegypti* (AF390098, AY432106) and *Ae. aegypti formosus* (AY056597) was downloaded from GenBank and combined with the sequences from this study. *Ae. albopictus* (MF148303) sequence was also included as an outgroup. The combined sequences were aligned as described earlier, and the alignment was used to infer a maximum likelihood phylogeny. Phylogenetic analysis was carried out in IQTREE [47] with simultaneous evaluation of the best model and tree inference being performed based on 1000 bootstrap replicates. The phylogenetic tree was visualized in FigTree v1.4.4 [48].

#### Vector competence data.

The Standard software package (***Stata***, version. 15.0; ***StataCorp***) was used to determine rates of infection (the proportion of mosquito bodies that were infected with CHIKV), dissemination (proportion of infected mosquitoes whose legs were positive for CHIKV) and transmission (proportion of infected mosquitoes whose bodies, legs and saliva were positive for CHIKV) in *Ae. aegypti* at 5-, 10- and 14-dpi (95% CI) for CHIKV. The same package was used to conduct a proportion test of differences to determine the statistical significance of infection, dissemination, and transmission rates between the challenged mosquito populations from the two study sites. The data for this study have been submitted as supporting document for this manuscript.

## Supporting information

S1 File *Ae. aegypti* CO1 study sequences and reference sequences ([*Aaa*; AF390098 & AY432106 & *Aaf*; AY056597]). Contains raw sequences of the study population’s CO1 genes and the reference CO1 genes used in the sequence alignment.
(PDF)

S2 File Vector competence data. Contains infection, dissemination and transmission results. (XLSX)

S3 File Plaque assays results. Contains how CHIKV titer was calculated. (DOCX)
