# Supplementary material for: Genetic diversity of Aedes aegypti populations from Kisumu and Busia counties, western Kenya, and their vector competence for chikungunya virus
Source: PLoS One. 2025 Mar 25;20(3):e0289191. doi: 10.1371/journal.pone.0289191 (PMC11936183; doi:10.1371/journal.pone.0289191)
Supplement: S1 File — Contains raw sequences of the study population’s CO1 genes and the reference CO1 genes used in the sequence alignment. (PDF) [file pone.0289191.s001.pdf]

>AF390098_Aedes_aegypti
TCGCGACAATGGTTATTTTCAACAAATCATAAAGATATTGGAACTTTATATTTCATTTTTGGAGTATGAT
CTGGAATAGTCGGAACTTCTCTAAGAATTTTAATTCGTGCTGAACTTAGCCACCCTGGTATATTTATTGG
GAATGACCAAATTTATAATGTAATTGTAACAGCTCATGCATTTATTATAATTTTCTTTATAGTAATGCCA
ATTATAATTGGAGGATTTGGAAATTGATTAGTTCCTTTAATATTAGGAGCCCCTGATATAGCTTTCCCTC
GAATGAATAATATAAGTTTTTGAATACTACCTCCTTCATTGACTCTTCTATTATCAAGCTCAATAGTAGA
AAATGGGGCAGGAACTGGGTGAACAGTTTATCCTCCTCTCTCTTCAGGAACAGCTCATGCTGGAGCTTCT
GTTGATTTAGCTATTTTTTCTCTTCATTTAGCTGGAATTTCCTCAATTTTAGGGGCAGTAAATTTTATTA
CAACTGTGATTAATATGCGATCGTCAGGGATTACTTTAGATCGACTACCCTTATTTGTTTGATCTGTAGT
TATTACAGCTATCTTATTACTTCTTTCTCTTCCTGTTTTAGCTGGAGCTATTACTATATTATTAACAGAC
CGAAACTTAAATACATCTTTCTTTGATCCAATCGGAGGGGGAGACCCTATTTTATACCAACACTTATTTT
GATTCTTTGGACACCCAGAAGTTTATATTTTAATTTTACCCGGATTTGGAATAATTTCTCATATTATTAC
TCAAGAAAGCGGAAAAAAGGAAACATTTGGAACTTTAGGAATAATTTATGCTATATTAACAATTGGATTA
TTGGGATTTATTGTTTGAGCTCATCATATATTTACAGTAGGTATAGACGTAGATACTCGAGCTTATTTTA
CTTCAGCAACTATAATTATTGCTGTTCCTACAGGAATTAAAATTTTTAGTTGATTAGCAACTTTACACGG
AACTCAATTAACATATAGTCCAGCCCTTCTATGATCATTAGGATTTGTATTTTTATTTACAGTTGGAGGT
TTAACAGGAGTAGTATTAGCTAATTCTTCAATTGATATTGTTCTTCATGATACTTATTACGTAGTTGCCC
ATTTTCATTACGTTTTATCTATAGGAGCTGTATTTGCTATTATAGCAGGATTTATTCATTGATACCCTTT
ATTAACAGGAATAGTTATAAACCCTTCATGATTAAAGGCTCAATTTAGTATAATATTTATTGGAGTAAAT
CTAACTTTCTTTCCTCAACATTTTTTAGGGTTAGCTGGAATACCTCGACGATACTCAGATTTTCCTGATA
GCTACTTAACTTGAAATATTATTTCTTCTTTAGGAAGAACAATTTCACTATTTGCCGTTATTTTCTTTTT
ATTTATTATTTGAGAAAGTATAATTACTCAACGAACACCTTCTTTCCCTATACAATTATCTTCATCTATT
GAATGATATCATACACTTCCTCCTGCAGAACATACTTATTCAGAACTACCACTACTTTCTTCTAATT
>AY432106_Aedes_aegypti
ATATCATTATTGAATATAAAGTTTGATTAAAAAGAATATTTTTCTTATATATAAATTTACAWWTTATCGC
CTAAACTTCAGCCATTTAATCGCGACAATGGTTATTTTCAACAAATCATAAAGATATTGGAACTTTATAT
TTCATTTTTGGAGTATGATCTGGAATAGTCGGAACTTCTCTAAGAATTTTAATTCGTGCTGAACTTAGCC
ACCCTGGTATATTTATTGGGAATGACCAAATTTATAATGTAATTGTAACAGCTCATGCATTTATTATAAT
TTTCTTTATAGTAATGCCAATTATAATTGGAGGATTTGGAAATTGATTAGTTCCTTTAATATTAGGAGCC
CCTGATATAGCTTTCCCTCGAATGAATAATATAAGTTTTTGAATACTACCTCCTTCATTGACTCTTCTAT
TATCAAGCTCAATAGTAGAAAATGGGGCAGGAACTGGGTGAACAGTTTATCCTCCTCTCTCTTCAGGAAC
AGCTCATGCTGGAGCTTCTGTTGATTTAGCTATTTTTTCTCTTCATTTAGCTGGAATTTCCTCAATTTTA
GGGGCAGTAAATTTTATTACAACTGTGATTAATATGCGATCGTCAGGGATTACTTTAGATCGACTACCCT
TATTTGTTTGATCTGTAGTTATTACAGCTATCTTATTACTTCTTTCTCTTCCTGTTTTAGCTGGAGCTAT
TACTATATTATTAACAGACCGAAACTTAAATACATCTTTCTTTGATCCAATCGGAGGGGGAGACCCTATT
TTATACCAACACTTATTTTGATTCTTTGGACACCCAGAAGTTTATATTTTAATTTTACCCGGATTTGGAA
TAATTTCTCATATTATTACTCAAGAAAGCGGAAAAAAGGAAACATTTGGAACTTTAGGAATAATTTATGC
TATATTAACAATTGGATTATTGGGATTTATTGTTTGAGCTCATCATATATTTACAGTAGGTATAGACGTA
GATACTCGAGCTTATTTTACTTCAGCAACTATAATTATTGCTGTTCCTACAGGAATTAAAATTTTTAGTT
GATTAGCAACTTTACACGGAACTCAATTAACATATAGTCCAGCCCTTCTATGATCATTAGGATTTGTATT
TTTATTTACAGTTGGAGGTTTAACAGGAGTAGTATTAGCTAATTCTTCAATTGATATTGTTCTTCATGAT
ACTTATTACGTAGTTGCCCATTTTCATTACGTTTTATCTATAGGAGCTGTATTTGCTATTATAGCAGGAT
TTATTCATTGATACCCTTTATTAACAGGAATAGTTATAAACCCTTCATGATTAAAGGCTCAATTTAGTAT
AATATTTATTGGAGTAAATCTAACTTTCTTTCCTCAACATTTTTTAGGGTTAGCTGGAATACCTCGACGA
TACTCAGATTTTCCTGATAGCTACTTAACTTGAAATATTATTTCTTCTTTAGGAAGAACAATTTCACTAT
TTGCCGTTATTTTCTTTTTATTTATTATTTGAGAAAGTATAATTACTCAACGAACACCTTCTTTCCCTAT
ACAATTATCTTCATCTATTGAATGATATCATACACTTCCTCCTGCAGAACATACTTATTCAGAATTACCA
CTACTTTCTTCTAATCBAAAAAAAAAAAAAAAAAAAAAAAAAAAAA
>AY056597_Aedes_aegypti_Formosus
TCGCGACAATGGTTATTTTCAACAAATCATAAAGATATTGGAACTTTATATTTCATTTTTGGAGTATGAT

CCGGAATAGTCGGAACTTCTTTAAGAATTTTAATTCGTGCTGAACTTAGCCACCCTGGTATATTTATTGG
GAATGACCAAATTTATAATGTAATTGTAACAGCTCATGCATTTATTATAATTTTCTTTATAGTAATACCA
ATTATAATTGGAGGATTTGGAAATTGATTAGTTCCTTTAATATTAGGAGCCCCTGATATAGCCTTTCCTC
GAATAAATAATATAAGTTTTTGAATACTACCTCCTTCATTGACTCTTCTATTATCAAGCTCAATAGTAGA
AAATGGGGCAGGAACTGGGTGAACAGTTTATCCTCCTCTCTCTTCAGGAACAGCTCATGCTGGAGCTTCT
GTTGATTTAGCTATTTTTTCTCTTCATTTAGCTGGAATTTCCTCAATTTTAGGGGCAGTAAATTTTATTA
CAACTGTAATTAATATACGATCGTCAGGAATTACTTTAGATCGACTACCCTTATTTGTTTGATCTGTAGT
TATTACAGCTATCTTATTACTTCTTTCTCTTCCTGTTTTAGCTGGGGCTATTACTATGTTATTAACAGAC
CGAAACTTAAATACATCTTTCTTTGATCCAATCGGAGGAGGAGACCCTATTTTATACCAACACTTATTCT
GATTCTTTGGACACCCAGAAGTTTATATTTTAATTTTACCCGGATTTGGAATAATTTCTCATATTATTAC
TCAAGAAAGTGGAAAAAAGGAAACATTTGGAACTTTAGGAATAATTTATGCTATATTAACAATTGGATTA
TTGGGATTTATTGTTTGAGCTCATCATATATTTACAGTAGGTATAGACGTAGATACTCGAGCTTATTTTA
CTTCAGCAACTATAATTATTGCTGTTCCTACAGGAATTAAAATTTTTAGTTGATTAGCAACTTTACACGG
AACTCAATTAACATATAGTCCAGCCCTTCTATGATCATTAGGATTTGTATTTTTATTTACAGTTGGAGGT
TTAACAGGAGTAGTATTAGCTAATTCTTCAATTGACATTGTTCTTCATGATACTTATTACGTAGTTGCCC
ATTTTCATTATGTTTTATCTATAGGAGCTGTATTTGCTATTATAGCAGGATTTATTCATTGATACCCTTT
ATTAACAGGAATAGTTATAAACCCTTCATGATTAAAGGCTCAATTTAGTATAATATTTATTGGAGTAAAT
CTAACTTTCTTTCCCCAACATTTTTTAGGATTAGCTGGAATACCTCGACGATACTCAGATTTTCCCGATA
GTTACTTAACTTGAAATATTATTTCTTCTTTAGGAAGAACAATTTCATTATTTGCCGTTATTTTCTTTTT
ATTTATTATTTGAGAAAGTATAATTACTCAACGAACACCTTCTTTCCCTATACAATTATCTTCATCTATT
GAATGATATCATACACTTCCTCCTGCAGAACATACTTATTCAGAATTACCACTACTTTCTTCTAATT
>MLB_Ae.aegpti_01
TAATTGAGAGGATTTAGAGAAATTAGATTAGTTCCTTTAATATTAGGAGCCCCTGATATA
GCTTTCCCTCGAATGAATAATATAAGTTTTTGAATACTACCTCCTTCATTGACTCTTCTA
TTATCAAGCTCAATAGTAGAAAATGGGGCAGGAACTGGGTGAACAGTTTATCCTCCTCTC
TCTTCAGGAACAGCTCATGCTGGAGCTTCTGTTGATTTAGCTATTTTTTCTCTTCATTTA
GCTGGAATTTCCTCAATTTTAGGGGCAGTAAATTTTATTACAACTGTGATTAATATACGA
TCGTCAGGGATTACTTTAGATCGACTACCCTTATTTGTTTGATCTGTAGTTATTACAGCT
ATCTTATTACTTCTTTCTCTTCCTGTTTTAGCTGGAGCTATTACTATATTATTAACAGAC
CGAAACTTAAATACATCTTTCTTTGATCCAATCGGAGGGGGAGACCCTATTTTATACCAA
CACTTATTTTGATTCTTTGGACACCCAGAAGTTTATATTTTAATTTTACCCGGATTTGGA
ATAATTTCTCATATTATTACTCAAGAAAGCGGAAAAAAGGAAACATTTGGAACTTTAGGA
ATAATTTATGCTATATTAACAATTGGATTATTAGGATTTATTGTTTGAGCTCATCATATA
TTTACAGTAGGTATAGACGTAGATACTCGAGCTTATTTTACTTCAGCAACTATAATTATT
GCTGTTCCTACAGGAATTAAAATTTTTAGTTGATTAGCAACTTTACACGGAACTCAATTA
ACATATAGTCCAGCCTCTTTTT
>MLB_Ae.aegpti_02
ATTATAATTGGAGGATTTGGAAATTAGATTAGTTCCTTTAATATTAGGAGCCCCTGATAT
AGCCTTTCCTCGAATAAATAATATAAGTTTTTGAATACTACCTCCTTCATTGACTCTTCT
ATTATCAAGCTCAATAGTAGAAAATGGGGCAGGAACTGGGTGAACAGTTTATCCTCCTCT
CTCTTCAGGAACAGCTCATGCTGGAGCTTCTGTTGATTTAGCTATTTTTTCTCTTCATTT
AGCTGGAATTTCCTCAATTTTAGGGGCAGTAAATTTTATTACAACTGTAATTAATATACG
ATCGTCAGGAATTACTTTAGATCGACTACCCTTATTTGTTTGATCTGTAGTTATTACAGC
TATCTTATTACTTCTTTCTCTTCCTGTTTTAGCTGGAGCTATTACTATATTATTAACAGA
CCGAAACTTAAATACATCTTTCTTTGATCCAATCGGAGGAGGAGATCCTATTTTATACCA
ACACTTATTCTGATTCTTTGGACACCCAGAAGTTTATATTTTAATTTTACCCGGATTTGG
AATAATTTCTCATATTATTACTCAAGAAAGTGGAAAAAAGGAAACATTTGGAACTTTAGG
AATAATTTATGCTATATTAACAATTGGATTATTGGGATTTATTGTTTGAGCTCATCATAT
ATTTACAGTAGGTATAGACGTAGATACTCGAGCTTATTTTACTTCAGCAACTATAATTAT
TGCTGTTCCTACAGGAATTAAAATTTTTAGTTGATTAGCAACTTTACACGGAACTCAATT

AACATATAGTCCAGCCTCTTTTTTT
>MLB_Ae.aegpti_03
TTATAATTGGAGGATTTGGAAATTGATTAGTTCCTTTAATATTAGGAGCCCCTGATATAG
CCTTCCCTCGAATAAATAATATAAGTTTTTGAATACTACCTCCTTCATTGACTCTTCTAT
TATCAAGCTCAATAGTAGAAAATGGGGCAGGAACTGGGTGAACAGTTTATCCTCCTCTCT
CTTCAGGAACAGCTCATGCTGGAGCTTCTGTTGATTTAGCTATTTTTTCTCTTCATTTAG
CTGGAATTTCCTCAATTTTAGGGGCAGTAAATTTTATTACAACTGTAATTAATATACGAT
CGTCAGGGATTACTTTAGATCGACTACCCTTATTTGTTTGATCTGTAGTTATTACAGCTA
TCTTATTACTTCTTTCTCTTCCTGTTTTAGCTGGAGCTATTACTATATTATTAACAGACC
GAAACTTAAATACATCTTTCTTTGATCCAATCGGAGGAGGAGACCCTATTTTATACCAAC
ACTTATTTTGATTCTTTGGGCACCCAGAAGTTTATATTTTAATTTTACCCGGATTTGGAA
TAATTTCTCATATTATTACTCAAGAAAGTGGAAAAAAGGAAACATTCGGAACTTTAGGAA
TAATTTATGCTATATTAACAATTGGATTATTGGGATTTATTGTTTGAGCTCATCATATAT
TTACAGTAGGTATAGACGTAGATACTCGAGCTTATTTTACTTCAGCAACTATAATTATTG
CTGTTCCTACAGGAATTAAAATTTTTAGTTGATTAGCAACTTTACACGGAACTCAATTAA
CATATAGTCCAGCTCT
>MLB_Ae.aegpti_04
TTAGATTAGTTCCTTTAATATTAGGAGCCCCTGATATAGCCTTTCCTCGAATAAATAATA
TAAGTTTTTGAATACTACCTCCTTCATTGACTCTTCTATTATCAAGCTCAATAGTAGAAA
ATGGAGCAGGAACTGGGTGAACAGTTTATCCTCCTCTCTCTTCAGGAACAGCTCATGCTG
GAGCTTCTGTTGATTTAGCTATTTTTTCTCTTCATTTAGCTGGAATTTCCTCAATTTTAG
GGGCAGTAAATTTTATTACAACTGTAATTAATATACGATCGTCAGGAATTACTTTAGATC
GACTACCCTTATTTGTTTGATCTGTAGTTATTACAGCTATCTTATTACTTCTTTCTCTTC
CTGTTTTAGCTGGAGCTATTACTATGTTATTAACAGACCGAAACTTAAATACATCTTTCT
TTGATCCAATCGGAGGAGGAGATCCTATTTTATACCAACACTTATTCTGATTCTTTGGAC
ACCCAGAAGTTTATATTTTAATTTTACCCGGATTTGGAATAATTTCTCATATTATTACTC
AAGAAAGTGGAAAAAAGGAAACATTTGGAACTTTAGGAATAATTTATGCTATATTAACAA
TTGGATTATTGGGATTTATTGTTTGAGCTCATCATATATTTACAGTAGGTATAGACGTAG
ATACTCGAGCTTATTTTACTTCAGCAACTATAATTATTGCTGTTCCTACAGGAATTAAAA
TTTTTAGTTGATTAGCAACTTTACACGGAACTCAATTAACATATAGTCCAGCCCTC
>MLB_Ae.aegpti_05
TATAATTGGAGGATTTGGAAATTAGATTAGTTCCTTTAATATTAGGAGCCCCTGATATAG
CCTTTCCTCGAATAAATAATATAAGTTTTTGAATACTACCTCCTTCATTGACTCTTCTAT
TATCAAGCTCAATAGTAGAAAATGGAGCAGGAACTGGGTGAACAGTTTATCCTCCTCTCT
CTTCAGGAACAGCTCATGCTGGAGCTTCTGTTGATTTAGCTATTTTTTCTCTTCATTTAG
CTGGAATTTCCTCAATTTTAGGGGCAGTAAATTTTATTACAACTGTAATTAATATACGAT
CGTCAGGAATTACTTTAGATCGACTACCCTTATTTGTTTGATCTGTAGTTATTACAGCTA
TCTTATTACTTCTTTCTCTTCCTGTTTTAGCTGGAGCTATTACTATGTTATTAACAGACC
GAAACTTAAATACATCTTTCTTTGACCCAATCGGAGGAGGAGATCCTATTTTATACCAAC
ACTTATTCTGATTCTTTGGACACCCAGAAGTTTATATTTTAATTTTACCCGGATTTGGAA
TAATTTCTCATATTATTACTCAAGAAAGTGGAAAAAAGGAAACATTTGGAACTTTAGGGA
TAATTTATGCTATATTAACAATTGGATTATTGGGATTTATTGTTTGAGCTCATCATATAT
TTACAGTAGGTATAGACGTAGATACTCGAGCTTATTTTACTTCAGCAACTATAATTATTG
CTGTTCCTACAGGAATTAAAATTTTTAGTTGATTAGCAACTTTACACGGAACTCAACTAA
CATATAGTCCAGCTCTTTTTGAGAATA
>MLB_Ae.aegpti_06
TAGTTCCTTTAATATTAGGAGCCCCTGATATAGCCTTTCCTCGAATAAATAATATAAGTT
TTTGAATACTACCTCCTTCATTGACTCTTCTATTATCAAGCTCAATAGTAGAAAATGGGG
CGGGAACTGGGTGAACAGTTTATCCTCCTCTCTCTTCAGGAACAGCTCATGCTGGAGCTT
CTGTTGATTTAGCTATTTTTTCTCTTCATTTAGCTGGAATTTCCTCAATTTTAGGGGCAG

TAAATTTTATTACAACTGTAATTAATATACGATCATCAGGAATTACTTTAGATCGACTAC
CCTTATTTGTTTGATCTGTAGTTATTACAGCTATCTTATTACTTCTTTCTCTTCCTGTTT
TAGCTGGAGCTATTACTATGTTATTAACAGACCGAAACTTAAATACATCTTTCTTTGATC
CAATCGGAGGAGGAGACCCTATTTTATACCAACACTTATTCTGATTCTTTGGACACCCAG
AAGTTTATATTTTAATTTTACCCGGATTTGGAATAATTTCTCATATTATTACTCAAGAAA
GTGGAAAAAAGGAAACATTTGGAACTTTAGGAATAATTTATGCTATATTAACAATTGGAT
TATTGGGATTTATTGTTTGAGCTCATCATATATTTACAGTAGGTATAGACGTAGATACTC
GAGCTTATTTTACTTCAGCAACTATAATTATTGCTGTTCCTACAGGAATTAAAATTTTTA
GTTGATTAGCAACTTTACACGGAACTCAATTAACATATAGTCCAGCCCTCCCATTGATAA
AAAAATTATTAA
>MLB_Ae.aegpti_07
ATTCCTTTAATATTAGGAGCCCCTGATATAGCCTTTCCTCGAATAAAAAATATAAGTTTT
TGAATACTACCTCCTTCATTGACTCTTCTATTATCAAGCTCAATAGTAGAAAATGGAGCA
GGAACTGGGTGAACAGTTTATCCTCCTCTCTTCAGGAACAGCTCACGCTGGAGCTTCT
GTTGATTTAGCTATTTTCACTCTCCATTTAGCTGGAATATCCCCACTTTTAGGGGGGGAA
AATTTTTTTACAACTGTGGTAAATATACAACCGTCGGGAAGTACTTTATATCAACTACCC
CTATTTGTTTGATCTGTAGTTATTACAGCTATCATATTACTTCTTTCTTCTCTTGTTGTT
TGAGCTGGTATTACTATGTTATTATCACACCAAAAAAAATTAACATTTTTTTTTGATCCT
CCCGGAGGAGGAAGAAATCTTTATTTATACCCACACTTATGCTGCTTGGGCCCCCCCCCC
TAAGTTTTTTTTTTTTTTTTAGCCCGGGGGATGGGATTCCTTTTTCCCATTATCACCCCA
GAAAAAGGGAAAAAAAGGAAAGTTTGGAAGTATTGAAAAGCTTTACGCCCTCTTAACTAG
TGGAGTATTTG
>MLB_Ae.aegpti_08
TAGTTCCTTTAATATTAGGAGCCCCTGATATAGCTTTCCCTCGAATGAATAATATAAGTT
TTTGAATACTACCTCCTTCATTGACTCTTCTATTATCAAGCTCAATAGTAGAAAATGGAG
CAGGAACTGGGTGAACAGTTTATCCTCCTCTCTCTTCAGGAACAGCTCACGCTGGAGCTT
CTGTTGATTTAGCTATTTTTTCTCTTCATTTAGCTGGAATTTCCTCAATTTTAGGGGCAG
TAAATTTTATTACAACTGTAATTAATATACGATCGTCAGGGATTACTTTAGATCGACTAC
CCTTATTTGTTTGATCTGTAGTTATTACAGCTATCTTATTACTTCTTTCTCTTCCTGTTT
TAGCCGGAGCTATTACTATATTATTAACAGACCGAAACTTAAATACATCTTTCTTTGACC
CAATCGGAGGGGGAGACCCTATTTTATACCAACACTTATTTTGATTCTTTGGACACCCAG
AAGTTTATATTTTAATTTTACCTGGATTTGGAATAATTTCTCATATTATTACTCAAGAAA
GTGGAAAAAAGGAAACATTTGGAACTTTAGGAATAATTTATGCTATATTAACAATTGGAT
TATTAGGATTTATTGTTTGAGCTCATCATATATTTACAGTAGGTATAGATGTAGATACTC
GAGCTTATTTTACTTCAGCAACTATAATTATTGCTGTTCCTACAGGAATTAAAATTTTTA
GTTGATTAGCAACTTTACACGGAACTCAATTAACATATAGTCCAGCCTCCCAATGAATC
>MLB_Ae.aegpti_09
TATAATTGAGAGGATTATGAGAAATTAGATTAGTTCCTTTAATATTAGGAGCCCCTGATA
TAGCTTTCCCTCGAATGAATAATATAAGTTTTTGAATACTACCTCCTTCATTGACTCTTC
TATTATCAAGCTCAATAGTAGAAAATGGAGCAGGAACTGGGTGAACAGTTTATCCTCCTC
TCTCTTCAGGAACAGCTCATGCTGGAGCTTCTGTTGATTTAGCTATTTTTTCTCTTCATT
TAGCTGGAATTTCCTCAATTTTAGGGGCAGTAAATTTTATTACAACTGTAATTAATATAC
GATCGTCAGGGATTACTTTAGATCGACTACCCTTATTTGTATGATCTGTAGTTATTACAG
CTATCTTATTACTTCTTTCTCTTCCTGTTTTAGCTGGAGCTATTACTATATTATTAACAG
ACCGAAACTTAAATACATCTTTCTTTGATCCAATCGGAGGGGGAGACCCTATTTTATACC
AACACTTATTTTGATTCTTTGGGCACCCAGAAGTTTATATTTTAATTTTACCCGGATTTG
GAATAATTTCTCATATTATTACTCAAGAAAGTGGAAAAAAGGAAACATTTGGAACTTTAG
GAATAATTTATGCTATATTAACAATTGGATTATTGGGATTTATTGTTTGAGCTCATCATA
TATTTACAGTAGGTATAGATGTAGATACTCGAGCTTATTTTACTTCAGCAACTATAATTA
TTGCTGTTCCTACAGGAATTAAAATTTTTAGTTGATTAGCAACTTTACACGGAACTCAAT

TAACATATAGTCCAGCCTCCCTTTAAAAAAAAAAAAAAAAAAAAA
>MLB_Ae.aegpti_10
ATTTCGGAGGATTATCGGAAATATAGATTAGTTCCTTTAATATTAGGAGCCCCTGATATA
GCCTTTTTCCTCGAATAAATAATATAAGTTTTTGAATACTACCTCCTTCATTGACTCTTC
TATTATCAAGCTCAATAGTAGAAAATGGAGCAGGAACTGGGTGAACAGTTTATCCTCCTC
TCTCTTCAGGAACAGCTCATGCTGGAGCTTCTGTTGATTTAGCTATTTTTTCTCTTCATT
TAGCTGGAATTTCCTCAATTTTAGGGGCAGTAAATTTTATTACAACTGTAATTAATATAC
GATCGTCAGGAATTACTTTAGATCGACTACCCTTATTTGTTTGATCTGTAGTTATTACAG
CTATCTTATTACTTCTTTCTCTTCCTGTTTTAGCTGGAGCTATTACTATGTTATTAACAG
ACCGAAACTTAAATACATCTTTCTTTGATCCAATCGGAGGAGGAGATCCTATTTTATACC
AACACTTATTCTGATTCTTTGGACACCCAGAAGTTTATATTTTAATTTTACCCGGATTTG
GAATAATTTCTCATATTATTACTCAAGAAAGTGGAAAAAAGGAAACATTTGGAACTTTAG
GAATAATTTATGCTATATTAACAATTGGATTATTGGGATTTATTGTTTGAGCTCATCATA
TATTTACAGTAGGTATAGACGTAGATACTCGAGCTTATTTTACTTCAGCAACTATAATTA
TTGCTGTTCCTACAGGAATTAAAATTTTTAGTTGATTAGCAACTTTACACGGAACTCAAT
TAACATATAGTCAGCTTTCTTAAAAAATATAAAAAAAAAAAAAAAAA
>MLB_Ae.aegpti_11
ATACCAATTATAATTGGAGGATTTGGAAATTGATTAGTTCCTTTAATATTAGGAGCCCCT
GATATAGCCTTTCCTCGAATAAATAATATAAGTTTTTGAATACTACCTCCTTCATTGACT
CTTCTATTATCAAGCTCAATAGTAGAAAATGGAGCAGGAACTGGGTGAACAGTTTATCCT
CCTCTCTCTTCAGGAACAGCTCATGCTGGGGCTTCTGTTGATTTAGCTATTTTTTCTCTT
CATTTAGCTGGAATTTCCTCAATTTTAGGGGCAGTAAATTTTATTACAACTGTAATTAAT
ATACGATCGTCAGGAATTACTTTAGATCGACTACCCTTATTTGTTTGATCTGTAGTTATT
ACAGCTATCTTATTACTTCTTTCTCTTCCTGTTTTAGCTGGAGCTATTACTATGTTATTA
ACAGACCGAAACTTAAATACATCTTTCTTTGATCCAATCGGAGGAGGAGATCCTATTTTA
TACCAACACTTATTCTGATTCTTTGGACACCCAGAAGTTTATATTTTAATTTTACCCGGA
TTTGGAATAATTTCTCATATTATTACTCAAGAAAGTGGAAAAAAGGAAACATTTGGAACT
TTAGGAATAATTTATGCTATATTAACAATTGGATTATTGGGATTTATTTGTTTGAGCTCA
TCATATATTTACAGTAGGTATAGACGTAGATACTCGAGCTTATTTTACTTCAGCAACTAT
AATTATTGCTGTTCCTACAGGAATTAAAATTTTTAGTTGATTAGCAACTTTACACGGAAC
TCAATTAACATATAGTCAGCTCCTTTTAAAAAAAAAAAAAAAAAAAAAA
>MLB_Ae.aegpti_12
TAATACCAATTATAATTGGAGGATTTGGAAATTGATTAGTTCCTTTAATATTAGGAGCCC
CTGATATAGCCTTTCCTCGAATAAATAATATAAGTTTTTGAATACTACCTCCTTCATTGA
CTCTTCTATTATCAAGCTCAATAGTAGAAAATGGAGCAGGAACTGGGTGAACAGTTTATC
CTCCTCTCTCTTCAGGAACAGCTCATGCTGGAGCTTCTGTTGATTTAGCTATTTTTTCTC
TTCATTTAGCTGGAATTTCCTCAATTTTAGGGGCAGTAAATTTTATTACAACTGTAATTA
ATATACGATCGTCAGGAATTACTTTAGATCGACTACCCTTATTTGTTTGATCTGTAGTTA
TTACAGCTATCTTATTACTTCTTTCTCTTCCTGTTTTAGCTGGAGCTATTACTATGTTAT
TAACAGACCGAAACTTAAATACATCTTTCTTTGATCCAATCGGAGGAGGAGATCCTATTT
TATACCAACACTTATTCTGATTCTTTGGACACCCAGAAGTTTATATTTTAATTTTACCCG
GATTTGGAATAATTTCTCATATTATTACTCAAGAAAGTGGAAAAAAGGAAACATTTGGAA
CTTTAGGAATAATTTATGCTATATTAACAATTGGATTATTGGGGTTTATTGTTTGAGCTC
ATCATATATTTACAGTAGGTATAGACGTAGATACTCGAGCTTATTTTACTTCAGCAACTA
TAATTATTGCTGTTCCTACAGGAATTAAGATTTTTAGTTGATTAGCAACTTTACACGGAA
CTCAATTAACATATAGTCCAGCTCTTTTTTCAATAATAAAAAAAAAAAAAAAAAAA
>MLB_Ae.aegpti_13
TTATAATTGGAGGATTTGGAAATTGATTAGTTCCTTTAATATTAGGAGCCCCTGATATAG
CCTTTCCTCGAATAAATAATATAAGTTTTTGAATACTACCTCCTTCATTGACTCTTCTAT
TATCAAGCTCAATAGTAGAAAATGGAGCAGGAACTGGGTGAACAGTTTATCCTCCTCTCT

CTTCAGGAACAGCTCATGCTGGGGCTTCTGTTGATTTAGCTATTTTTTCTCTTCATTTAG
CTGGAATTTCCTCAATTTTAGGGGCAGTAAATTTTATTACAACTGTAATTAATATACGAT
CGTCAGGAATTACTTTAGATCGACTACCCTTATTTGTTTGATCTGTAGTTATTACAGCTA
TCTTATTACTTCTTTCTCTTCCTGTTTTAGCTGGAGCTATTACTATGTTATTAACAGACC
GAAACTTAAATACATCTTTCTTTGATCCAATCGGAGGAGGAGATCCTATTTTATACCAAC
ACTTATTCTGATTCTTTGGACACCCAGAAGTTTATATTTTAATTTTACCCGGATTTGGAA
TAATTTCTCATATTATTACTCAAGAAAGTGGAAAAAAGGAAACATTTGGAACTTTAGGAA
TAATTTATGCTATATTAACAATTGGATTATTGGGGTTTATTGTTTGAGCTCATCATATAT
TTACAGTAGGTATAGACGTAGATACTCGAGCTTATTTTACTTCAGCAACTATAATTATTG
CTGTTCCTACAGGAATTAAAATTTTTAGTTGATTAGCAACTTTACACGGAACTCAATTAA
CATATAGTCCAGCTCTCTT
>MLB_Ae.aegpti_14
ATGCCATTATAATTGGAGGATTTGGAAATTGATTAGTTCCTTTAATATTAGGAGCCCCTG
ATATAGCTTTCCCTCGAATGAATAATATAAGTTTTTGAATACTACCTCCTTCATTGACTC
TTCTATTATCAAGCTCAATAGTAGAAAATGGAGCAGGAACTGGGTGAACAGTTTATCCTC
CTCTCTCTTCAGGAACAGCTCATGCTGGAGCTTCTGTTGATTTAGCTATTTTTTCTCTTC
ATTTAGCTGGAATTTCCTCAATTTTAGGAGCAGTAAATTTTATTACAACTGTAATTAATA
TACGATCGTCAGGGATTACTTTAGATCGACTACCCTTATTTGTATGATCTGTAGTTATTA
CAGCTATCTTATTACTTCTTTCTCTTCCTGTTTTAGCTGGAGCTATTACTATATTATTAA
CAGACCGAAACTTAAATACATCTTTCTTTGATCCAATCGGAGGGGGAGACCCTATTTTAT
ACCAACACTTATTTTGATTCTTTGGGCACCCAGAAGTTTATATTTTAATTTTACCCGGAT
TTGGAATAATTTCTCATATTATTACTCAAGAAAGTGGAAAAAAGGAAACATTTGGAACTT
TAGGAATAATTTATGCTATATTAACAATTGGATTATTGGGATTTATTGTTTGAGCTCATC
ATATATTTACAGTAGGTATAGATGTAGATACTCGAGCTTATTTTACTTCAGCAACTATAA
TTATTGCTGTTCCTACAGGAATTAAAATTTTTAGTTGATTAGCAACTTTACACGGAACTC
AATTAACATATAGTCCAGCTCTC
>MLB_Ae.aegpti_15
ATACCTATTATAATTGGAGGATTTGGAAATTAGATTAGTTCCTTTAATATTAGGAGCCCC
TGATATAGCCTTTCCTCGAATAAATAATATAAGTTTTTGAATACTACCTCCTTCATTGAC
TCTTCTATTATCAAGCTCAATAGTAGAAAATGGAGCAGGAACTGGGTGAACAGTTTATCC
TCCTCTCTCTTCAGGAACAGCTCATGCTGGAGCTTCTGTTGATTTAGCTATTTTTTCTCT
TCATTTAGCTGGAATTTCCTCAATTTTAGGGGCAGTAAATTTTATTACAACTGTAATTAA
TATACGATCGTCAGGAATTACTTTAGATCGACTACCCTTATTTGTTTGATCTGTAGTTAT
TACAGCTATCTTATTACTTCTTTCTCTTCCTGTTTTAGCTGGAGCTATTACTATGTTATT
AACAGACCGAAACTTAAATACATCTTTCTTTGATCCAATCGGAGGAGGAGATCCTATTTT
ATACCAACACTTATTCTGATTCTTTGGACACCCAGAAGTTTATATTTTAATTTTACCCGG
ATTTGGAATAATTTCTCATATTATTACTCAAGAAAGTGGAAAAAAGGAAACATTTGGAAC
TTTAGGAATAATTTATGCTATATTAACAATTGGATTATTGGGATTTATTGTTTGAGCTCA
TCATATATTTACAGTAGGTATAGACGTAGATACTCGAGCTTATTTTACTTCAGCAACTAT
AATTATTGCTGTTCCTACAGGAATTAAAATTTTTAGTTGATTAGCAACTTTACACGGAAC
TCAATTAACATATAGTCCAGCCCTC
>MLB_Ae.aegpti_16
TTTATAATTTAGGAGGATTTTGGAAATTGATTAGTTCCTTTAATATTAGGAGCCCCTGAT
ATAGCCTTTCCTCGAATAAATAATATAAGTTTTTGAATACTACCTCCTTCATTGACTCTT
CTATTATCAAGCTCAATAGTAGAAAATGGAGCAGGAACTGGGTGAACAGTTTATCCTCCT
CTCTCTTCAGGAACAGCTCATGCTGGAGCTTCTGTTGATTTAGCTATTTTTTCTCTTCAT
TTAGCTGGAATTTCCTCAATTTTAGGGGCAGTAAATTTTATTACAACTGTAATTAATATA
CGATCGTCAGGAATTACTTTAGATCGACTACCCTTATTTGTTTGATCTGTAGTTATTACA
GCTATCTTATTACTTCTTTCTCTTCCTGTTTTAGCTGGAGCTATTACTATGTTATTAACA
GACCGAAACTTAAATACATCTTTCTTTGATCCAATCGGAGGAGGAGATCCTATTTTATAC

CAACACTTATTCTGATTCTTTGGACACCCAGAAGTTTATATTTTAATTTTACCCGGATTT
GGAATAATTTCTCATATTATTACTCAAGAAAGTGGAAAAAAGGAAACGTTTGGAACTTTA
GGAATAATTTATGCTATATTAACAATTGGATTATTGGGATTTATTGTTTGAGCTCATCAT
ATATTTACAGTAGGTATAGACGTAGATACTCGAGCTTATTTTACTTCAGCAACTATAATT
ATTGCTGTTCCTACAGGAATTAAAATTTTTAGTTGATTAGCAACTTTACACGGAACTCAA
TTAACATATAGTCAGCCCCCTTCAAAAAATAAAAAAAAAAAAAAAAAAA
>MLB_Ae.aegpti_17
CCATTATAATTGGAGGATTTGGAAATTGATTAGTTCCTTTAATATTAGGAGCCCCTGATA
TAGCCTTTCCTCGAATAAATAATATAAGTTTTTGAATACTACCTCCTTCATTGACTCTTC
TATTATCAAGCTCAATAGTAGAAAATGGAGCAGGAACTGGGTGAACAGTTTATCCTCCTC
TCTCTTCAGGAACAGCTCATGCTGGAGCTTCTGTTGATTTAGCTATTTTTTCTCTTCATT
TAGCTGGAATTTCTTCAATTTTAGGGGCAGTAAATTTTATTACAACTGTAATTAATATAC
GATCGTCAGGAATTACTTTAGATCGACTACCCCTATTTGTTTGATCTGTAGTTATTACAG
CTATTTTATTACTTCTTTCTCTTCCTGTTTTAGCTGGAGCTATTACTATATTATTAACAG
ACCGAAACTTAAATACATCTTTCTTTGATCCAATCGGAGGAGGAGACCCTATTTTATACC
AACACTTATTCTGATTCTTTGGACACCCAGAAGTTTATATTTTAATTTTACCCGGATTTG
GAATAATTTCTCATATTATTACTCAAGAAAGTGGAAAAAAGGAAACATTTGGAACTTTAG
GAATAATTTATGCTATATTAACAATTGGATTATTGGGATTTATTGTTTGAGCTCATCATA
TATTTACAGTAGGTATAGACGTAGATACTCGAGCTTATTTTACTTCAGCAACTATAATTA
TTGCTGTTCCTACAGGAATTAAAATTTTTAGTTGATTAGCAACTCTACACGGAACTCAAT
TAACATATAGTCCAGCCCTTTTT
>MLB_Ae.aegpti_18
CCAATTATAATTGGAGGATTTGGAAATTGATTAGTTCCTTTAATATTAGGAGCCCCTGAT
ATAGCCTTTCCTCGAATAAATAATATAAGTTTTTGAATACTACCTCCTTCATTGACTCTT
CTATTATCAAGCTCAATAGTAGAAAATGGAGCAGGAACTGGGTGAACAGTTTATCCTCCT
CTCTCTTCAGGAACAGCTCATGCTGGAGCTTCTGTTGATTTAGCTATTTTTTCTCTTCAT
TTAGCTGGAATTTCCTCAATTTTAGGGGCAGTAAATTTTATTACAACTGTAATTAATATA
CGATCGTCAGGAATTACTTTAGATCGACTACCCTTATTTGTTTGATCTGTAGTTATTACA
GCTATCTTATTACTTCTTTCTCTTCCTGTTTTAGCTGGAGCTATTACTATGTTATTAACA
GACCGAAACTTAAATACATCTTTCTTTGATCCAATCGGAGGAGGAGATCCTATTTTATAC
CAACACTTATTCTGATTCTTTGGACACCCAGAAGTTTATATTTTAATTTTACCCGGATTT
GGAATAATTTCTCATATTATTACTCAAGAAAGTGGAAAAAAGGAAACATTTGGAACTTTA
GGAATAATTTATGCTATATTAACAATTGGATTATTGGGGTTTATTGTTTGAGCTCATCAT
ATATTTACAGTAGGTATAGACGTAGATACTCGAGCTTATTTTACTTCAGCAACTATAATT
ATTGCTGTTCCTACAGGAATTAAAATTTTTAGTTGATTAGCAACTTTACACGGAACTCAA
TTAACATATAGTCAGCTCCCTTAAGAAGA
>MLB_Ae.aegpti_19
ATACCTATTATAATTGGAGGATTTGGAAATTGATTAGTTCCTTTAATATTAGGAGCCCCT
GATATAGCCTTTCCTCGAATAAATAATATAAGTTTTTGAATACTACCTCCTTCATTGACT
CTTCTATTATCAAGCTCAATAGTAGAAAATGGGGCAGGAACTGGGTGAACAGTTTATCCT
CCTCTCTCTTCAGGAACAGCTCATGCTGGAGCTTCTGTTGATTTAGCTATTTTTTCTCTT
CATTTAGCTGGAATTTCCTCAATTTTAGGGGCAGTAAATTTTATTACAACTGTAATTAAT
ATACGATCGTCAGGAATTACTTTAGATCGACTACCCTTATTTGTTTGATCTGTAGTTATT
ACAGCTATCTTATTACTTCTTTCTCTTCCTGTTTTAGCTGGAGCTATTACTATGTTATTA
ACAGACCGAAACTTAAATACATCTTTCTTTGATCCAATCGGAGGAGGAGATCCTATTTTA
TACCAACACTTATTCTGATTCTTTGGACACCCAGAAGTTTATATTTTAATTTTACCCGGA
TTTGGAATAATTTCTCATATTATTACTCAAGAAAGTGGAAAAAAGGAAACATTTGGAACT
TTAGGAATAATTTATGCTATATTAACAATTGGATTATTGGGATTTATTGTTTGAGCTCAT
CATATATTTACAGTAGGTATAGACGTAGATACTCGAGCTTATTTTACTTCAGCAACTATA
ATTATTGCTGTTCCTACAGGAATTAAAATTTTTAGTTGATTAGCAACTTTACACGGAACT

CAATTAACATATAGTCCAGCCTCTCCTTTTAAAACATA
>MLB_Ae.aegpti_20
TATAATTGGAGGATTTGGAAATTAGATTAGTTCCTTTAATATTAGGAGCCCCTGATATAG
CCTTTCCTCGAATAAATAATATAAGTTTTTGAATACTACCTCCTTCATTGACTCTTCTAT
TATCAAGCTCAATAGTAGAAAATGGGGCAGGAACTGGGTGAACAGTTTATCCTCCTCTCT
CTTCAGGAACAGCTCATGCTGGAGCTTCTGTTGATTTAGCTATTTTTTCTCTTCATTTAG
CTGGAATTTCCTCAATTTTAGGGGCAGTAAATTTTATTACAACTGTAATTAATATACGAT
CGTCAGGAATTACTTTAGATCGACTACCCTTATTTGTTTGATCTGTAGTTATTACAGCTA
TCTTATTACTTCTTTCTCTTCCTGTTTTAGCTGGAGCTATTACTATGTTATTAACAGACC
GAAACTTAAATACATCTTTCTTTGATCCAATCGGAGGAGGAGATCCTATTTTATACCAAC
ACTTATTCTGATTCTTTGGACACCCAGAAGTTTATATTTTAATTTTACCCGGATTTGGAA
TAATTTCTCATATTATTACTCAAGAAAGTGGAAAAAAGGAAACATTTGGAACTTTAGGAA
TAATTTATGCTATATTAACAATTGGATTATTGGGATTTATTGTTTGAGCTCATCATATAT
TTACAGTAGGTATAGACGTAGATACTCGAGCTTATTTTACTTCAGCAACTATAATTATTG
CTGTTCCTACAGGAATTAAAATTTTTAGTTGATTAGCAACTTTACACGGAACTCAATTAA
CATATAGTCCAGCCTCCCCAAA
>MLB_Ae.aegpti_21
TAGTTCCTTTAATATTAGGAGCCCCTGATATAGCCTTTCCTCGAATAAATAATATAAGTT
TTTGAATACTACCTCCTTCATTGACTCTTCTATTATCAAGCTCAATAGTAGAAAATGGAG
CAGGAACTGGGTGAACAGTTTATCCTCCTCTCTCTTCAGGAACAGCTCATGCTGGAGCTT
CTGTTGATTTAGCTATTTTTTCTCTTCATTTAGCTGGAATTTCCTCAATTTTAGGGGCAG
TAAATTTTATTACAACTGTAATTAATATACGATCGTCAGGAATTACTTTAGATCGACTAC
CCTTATTTGTTTGATCTGTAGTTATTACAGCTATCTTATTACTTCTTTCTCTTCCTGTTT
TAGCTGGAGCTATTACTATGTTATTAACAGACCGAAACTTAAATACATCTTTCTTTGATC
CAATCGGAGGAGGAGACCCTATTTTATACCAACACTTATTCTGATTCTTTGGACACCCAG
AAGTTTATATTTTAATTTTACCCGGATTTGGAATAATTTCTCATATTATTACTCAAGAAA
GTGGAAAAAAGGAAACATTTGGAACTTTAGGAATAATTTATGCTATATTAACAATTGGAT
TATTGGGATTTATTGTTTGAGCTCATCATATATTTACAGTAGGTATAGACGTAGATACTC
GAGCTTATTTTACTTCAGCAACTATAATTATTGCTGTTCCTACAGGAATTAAAATTTTTA
GTTGATTAGCAACTTTACACGGAACTCAATTAACATATAGTCCAGCCCTCTTAATGGATC
ATAA
>MLB_Ae.aegpti_22
TATAATTGAGAGGATTTGAGAAATTAGATTAGTTCCTTTAATATTAGGAGCCCCTGATAT
AGCTTTTCCTCGAATAAATAATATAAGTTTTTGAATACTACCTCCTTCATTGACTCTTCT
ATTATCAAGCTCAATAGTAGAAAATGGGGCAGGAACTGGGTGAACAGTTTATCCTCCTCT
CTCTTCAGGAACAGCTCATGCTGGAGCTTCTGTTGATTTAGCTATTTTTTCTCTTCATTT
AGCTGGAATTTCCTCAATTTTAGGAGCAGTAAATTTTATTACAACTGTAATTAATATACG
ATCGTCAGGAATTACTTTAGATCGACTACCCTTATTTGTTTGATCTGTAGTTATTACAGC
TATCTTATTACTTCTTTCTCTTCCTGTTTTAGCTGGAGCTATTACTATGTTATTAACAGA
CCGAAACTTAAATACATCTTTCTTTGACCCAATCGGAGGAGGAGATCCTATTTTATACCA
ACACTTATTCTGATTCTTTGGACACCCAGAAGTTTATATTTTAATTTTACCCGGATTTGG
AATAATTTCTCATATTATTACTCAAGAAAGTGGAAAAAAGGAAACATTTGGAACTTTAGG
AATAATTTATGCTATATTAACAATTGGATTATTGGGATTTATTGTTTGAGCTCATCATAT
ATTTACAGTAGGTATAGACGTAGATACTCGAGCTTATTTTACTTCAGCAACTATAATTAT
TGCTGTTCCTACAGGAATTAAAATTTTTAGTTGATTAGCAACTTTACACGGAACTCAATT
AACATATAGTCCAGCCTCT
>MLB_Ae.aegpti_23
TTATAATTGGAGGATTTGGAAATTGATTAGTTCCTTTAATATTAGGAGCCCCTGATATAG
CCTTTCCTCGAATAAATAATATAAGTTTTTGAATACTACCTCCTTCATTGACTCTTCTAT
TATCAAGCTCAATAGTAGAAAATGGAGCAGGAACTGGGTGAACAGTTTATCCTCCTCTCT

CTTCAGGAACAGCTCATGCTGGAGCTTCTGTTGATTTAGCTATTTTTTCTCTTCATTTAG
CTGGAATTTCCTCAATTTTAGGGGCAGTAAATTTTATTACAACTGTAATTAATATACGAT
CGTCAGGAATTACTTTAGATCGACTACCCTTATTTGTTTGATCTGTAGTTATTACAGCTA
TCTTATTACTTCTTTCTCTTCCTGTTTTAGCTGGAGCTATTACTATGTTATTAACAGACC
GAAACTTAAATACATCTTTCTTTGATCCAATCGGAGGAGGAGATCCTATTTTATACCAAC
ACTTATTCTGATTCTTTGGACACCCAGAAGTTTATATTTTAATTTTACCCGGATTTGGAA
TAATTTCTCATATTATTACTCAAGAAAGTGGAAAAAAGGAAACATTTGGAACTTTAGGAA
TAATTTATGCTATATTAACAATTGGATTATTGGGATTTATTGTTTGAGCTCATCATATAT
TTACAGTAGGTATAGACGTAGATACTCGAGCTTATTTTACTTCAGCAACTATAATTATTG
CTGTTCCTACAGGAATTAAAATTTTTAGTTGATTAGCAACTTTACACGGAACTCAATTAA
CATATAGTCCAGCCTCTCTTTTA
>MLB_Ae.aegpti_24
TGGAGGATTTGGAAATTAGATTAGTTCCTTTAATATTAGGAGCCCCTGATATAGCCTTTC
CTCGAATAAATAATATAAGTTTTTGAATACTACCTCCTTCATTGACTCTTCTATTATCAA
GCTCAATAGTAGAAAATGGAGCAGGAACTGGGTGAACAGTTTATCCTCCTCTCTCTTCAG
GAACAGCTCATGCTGGAGCTTCTGTTGATTTAGCTATTTTTTCTCTTCATTTAGCTGGAA
TTTCCTCAATTTTAGGGGCAGTAAATTTTATTACAACTGTAATTAATATACGATCGTCAG
GAATTACTTTAGATCGACTACCCTTATTTGTTTGATCTGTAGTTATTACAGCTATCTTAT
TACTTCTTTCTCTTCCTGTTTTAGCTGGAGCTATTACTATGTTATTAACAGACCGAAACT
TAAATACATCTTTCTTTGATCCAATCGGAGGAGGAGATCCTATTTTATACCAACACTTAT
TCTGATTCTTTGGACACCCAGAAGTTTATATTTTAATTTTACCCGGATTTGGAATAATTT
CTCATATTATTACTCAAGAAAGTGGAAAAAAGGAAACATTTGGAACTTTAGGAATAATTT
ATGCTATATTAACAATTGGATTATTGGGATTTATTGTTTGAGCTCATCATATATTTACAG
TAGGTATAGACGTAGATACTCGAGCTTATTTTACTTCAGCAACTATAATTATTGCTGTTC
CTACAGGAATTAAAATTTTTAGTTGATTAGCAACTTTACACGGAACTCAATTAACATATA
GTCCAGCCCTCCCA
>MLB_Ae.aegpti_25
TTAGATTAGTTCCTTTAATATTAGGAGCCCCTGATATAGCCTTTCCTCGAATAAATAATA
TAAGTTTTTGAATACTACCTCCTTCATTGACTCTTCTATTATCAAGCTCAATAGTAGAAA
ATGGAGCAGGAACTGGGTGAACAGTTTATCCTCCTCTCTCTTCAGGAACAGCTCATGCTG
GAGCTTCTGTTGATTTAGCTATTTTTTCTCTTCATTTAGCTGGAATTTCCTCAATTTTAG
GGGCAGTAAATTTTATTACAACTGTAATTAATATACGATCGTCAGGAATTACTTTAGATC
GACTACCCTTATTTGTTTGATCTGTAGTTATTACAGCTATCTTATTACTTCTTTCTCTTC
CTGTTTTAGCTGGAGCTATTACTATGTTATTAACAGACCGAAACTTAAATACATCTTTCT
TTGATCCAATCGGAGGAGGAGATCCTATTTTATACCAACACTTATTCTGATTCTTTGGAC
ACCCAGAAGTTTATATTTTAATTTTACCCGGATTTGGAATAATTTCTCATATTATTACTC
AAGAAAGTGGAAAAAAGGAAACATTTGGAACTTTAGGAATAATTTATGCTATATTAACAA
TTGGATTATTGGGATTTATTGTTTGAGCTCATCATATATTTACAGTAGGTATAGACGTAG
ATACTCGAGCTTATTTTACTTCAGCAACTATAATTATTGCTGTTCCTACAGGAATTAAAA
TTTTTAGTTGATTAGCAACTTTACACGGAACTCAATTAACATATAGTCCGACCTCCCCTT
GATCAAAAAAATATTAAAGGGGAAGTTGGAACACTGCAGGAGTGGT
>MLB_Ae.aegpti_26
AGGATTATGAGTAAATAAGATTAGTTCCTTTAATATTAGGAGCCCCTGATATAGCTTTCC
CTCGAATGAATAATATAAGTTTTTGAATACTACCTCCTTCATTGACTCTTCTATTATCAA
GCTCAATAGTAGAAAATGGAGCAGGAACTGGGTGAACAGTTTATCCTCCTCTCTCTTCAG
GAACAGCTCATGCTGGAGCTTCTGTTGATTTAGCTATTTTTTCTCTTCATTTAGCTGGAA
TTTCCTCAATTTTAGGGGCAGTAAATTTTATTACAACTGTAATTAATATACGATCGTCAG
GGATTACTTTAGATCGACTACCCTTATTTGTTTGATCTGTAGTTATTACAGCTATCTTAT
TACTTCTTTCTCTTCCTGTTTTAGCTGGAGCTATTACTATATTATTAACAGACCGAAACT
TAAATACATCTTTCTTTGATCCAATCGGAGGGGGAGACCCTATTTTATACCAACACTTAT

TTTGATTCTTTGGACACCCAGAAGTTTATATTTTAATTTTACCCGGATTTGGAATAATTT
CTCATATTATTACTCAAGAAAGTGGAAAAAAGGAAACATTTGGAACTTTAGGAATAATTT
ATGCTATATTAACAATTGGATTATTGGGATTTATTGTTTGAGCTCATCATATATTTACAG
TAGGTATAGATGTAGATACTCGAGCTTATTTTACTTCAGCAACTATAATTATTGCTGTTC
CTACAGGAATTAAAATTTTTAGTTGATTAGCAACCTTACACGGAACTCAATTAACATATA
GTCCAGCCTTTTT
>MLB_Ae.aegpti_27
AGTTCCTTTAATATTAGGAGCCCCTGATATAGCCTTTCCTCGAATAAATAATATAAGTTT
TTGAATACTACCTCCTTCATTGACTCTTCTATTATCAAGCTCAATAGTAGAAAATGGAGC
AGGAACTGGGTGAACAGTTTATCCTCCTCTCTCTTCAGGAACAGCTCATGCTGGAGCTTC
TGTTGATTTAGCTATTTTTTCTCTTCATTTAGCTGGAATTTCCTCAATTTTAGGGGCAGT
AAATTTTATTACAACTGTAATTAATATACGATCGTCAGGAATTACTTTAGATCGACTACC
CTTATTTGTTTGATCTGTAGTTATTACAGCTATCTTATTACTTCTTTCTCTTCCTGTTTT
AGCTGGAGCTATTACTATGTTATTAACAGACCGAAACTTAAATACATCTTTCTTTGATCC
AATCGGAGGAGGAGATCCTATTTTATACCAACACTTATTCTGATTCTTTGGACACCCAGA
AGTTTATATTTTAATTTTACCCGGATTTGGAATAATTTCTCATATTATTACTCAAGAAAG
TGGAAAAAAGGAAACATTTGGAACTTTAGGAATAATTTATGCTATATTAACAATTGGATT
ATTGGGATTTATTGTTTGAGCTCATCATATATTTACAGTAGGTATAGACGTAGATACTCG
AGCTTATTTTACTTCAGCAACTATAATTATTGCTGTTCCTACAGGAATTAAAATTTTTAG
TTGATTAGCAACTTTACACGGAACTCAATTAACATATAGTCCAGCCCTCCATTGA
>MLB_Ae.aegpti_28
TGGAGGATTATGAGAAATTAGATTAGTTCCTTTAATATTAGGAGCCCCTGATATAGCCTT
TCCTCGAATAAATAATATAAGTTTTTGAATACTACCTCCTTCATTGACTCTTCTATTATC
AAGCTCAATAGTAGAAAATGGAGCAGGAACTGGGTGAACAGTTTATCCTCCTCTCTCTTC
AGGAACAGCTCATGCTGGAGCTTCTGTTGATTTAGCTATTTTTTCTCTTCATTTAGCTGG
AATTTCCTCAATTTTAGGGGCAGTAAATTTTATTACAACTGTAATTAATATACGATCGTC
AGGAATTACTTTAGATCGACTACCCTTATTTGTTTGATCTGTAGTTATTACAGCTATCTT
ATTACTTCTTTCTCTTCCTGTTTTAGCTGGAGCTATTACTATGTTATTAACAGACCGAAA
CTTAAATACATCTTTCTTTGATCCAATCGGAGGAGGAGATCCTATTTTATACCAACACTT
ATTCTGATTCTTTGGACACCCAGAAGTTTATATTTTAATTTTACCCGGATTTGGAATAAT
TTCTCATATTATTACTCAAGAAAGTGGAAAAAAGGAAACATTTGGAACTTTAGGAATAAT
TTATGCTATATTAACAATTGGATTATTGGGATTTATTGTTTGAGCTCATCATATATTTAC
AGTAGGTATAGACGTAGATACTCGAGCTTATTTTACTTCAGCAACTATAATTATTGCTGT
TCCTACAGGAATTAAAATTTTTAGTTGATTAGCAACTTTACACGGAACTCAATTAACATA
TAGTCCAGCCTCTTTTTT
>MLB_Ae.aegpti_29
ATTTCGGAGGATTATGGAAATTTCGATTAGTTCCTTTAATATTAGGAGCCCCTGATATAG
CCTTCTCCTCGAATAAATAATATAAGTTTTTGAATACTACCTCCTTCATTGACTCTTCTA
TTATCAAGCTCAATAGTAGAAAATGGAGCAGGAACTGGATGAACAGTTTATCCTCCTCTC
TCTTCAGGAACAGCTCATGCTGGAGCTTCTGTTGATTTAGCTATTTTTTCTCTTCATTTA
GCTGGAATTTCCTCAATTTTAGGGGCAGTAAATTTTATTACAACTGTAATTAATATACGA
TCGTCAGGAATTACTTTAGATCGACTACCCTTATTTGTTTGATCTGTAGTTATTACAGCT
ATCTTATTACTTCTTTCTCTTCCTGTTTTAGCTGGAGCTATTACTATGTTATTAACAGAC
CGAAACTTAAATACATCTTTCTTTGATCCAATCGGAGGAGGAGATCCTATTTTATACCAA
CACTTATTCTGATTCTTTGGACACCCAGAAGTTTATATTTTAATTTTACCCGGATTTGGA
ATAATTTCTCATATTATTACTCAAGAAAGTGGAAAAAAGGAAACATTTGGAACTTTAGGA
ATAATTTATGCTATATTAACAATTGGATTATTGGGATTTATTGTTTGAGCTCATCATATA
TTTACAGTAGGTATAGACGTAGATACTCGAGCTTATTTTACTTCAGCAACTATAATTATT
GCTGTTCCTACAGGAATTAAAATTTTTAGTTGATTAGCAACTTTACACGGAACTCAATTA
ACATATAGTCAGCTTTTTTTAATAA

>MLB_Ae.aegpti_30
TGGAGGATTTATGGAAATATAGATTAGTTCCTTTAATATTAGGAGCCCCTGATATAGCTT
TTCCTCGAATAAATAATATAAGTTTTTGAATACTACCTCCTTCATTGACTCTTCTATTAT
CAAGCTCAATAGTAGAAAATGGGGCAGGAACTGGGTGAACAGTTTATCCTCCTCTCTCTT
CAGGAACAGCTCATGCTGGAGCTTCTGTTGATTTAGCTATTTTTTCTCTTCATTTAGCTG
GAATTTCCTCAATTTTAGGGGCAGTAAATTTTATTACAACTGTAATTAATATACGATCGT
CAGGAATTACTTTAGATCGACTACCCTTATTTGTTTGATCTGTAGTTATTACAGCTATCT
TATTACTTCTTTCTCTTCCTGTTTTAGCTGGAGCTATTACTATGTTATTAACAGACCGAA
ACTTAAATACATCTTTCTTTGATCCAATCGGAGGAGGAGATCCTATTTTATACCAACACT
TATTCTGATTCTTTGGACACCCAGAAGTTTATATTTTAATTTTACCCGGATTTGGAATAA
TTTCTCATATTATTACTCAAGAAAGTGGAAAAAAGGAAACATTTGGAACTTTAGGAATAA
TTTATGCTATATTAACAATTGGATTATTGGGATTTATTGTTTGAGCTCATCATATATTTA
CAGTAGGTATAGACGTAGATACTCGAGCTTATTTTACTTCAGCAACTATAATTATTGCTG
TTCCTACAGGAATTAAAATTTTTAGTTGATTAGCAACTTTACACGGAACTCAATTAACAT
ATAGTCCAGCCTCTTTTT
>MLB_Ae.aegpti_31
TAATTTCGGAGGATTATCGGAAATTTCGATTAGTTCCTTTAATATTAGGAGCCCCTGATA
TAGCTTTTTCCTCGAATAAATAATATAAGTTTTTGAATACTACCTCCTTCATTGACTCTT
CTATTATCAAGCTCAATAGTAGAAAATGGAGCAGGAACTGGGTGAACAGTTTATCCTCCT
CTCTCTTCAGGAACAGCTCATGCTGGAGCTTCTGTTGATTTAGCTATTTTTTCTCTTCAT
TTAGCTGGAATTTCCTCAATTTTAGGGGCAGTAAATTTTATTACAACTGTAATTAATATA
CGATCGTCAGGAATTACTTTAGATCGACTACCCTTATTTGTTTGATCTGTAGTTATTACA
GCTATCTTATTACTTCTTTCTCTTCCTGTTTTAGCTGGAGCTATTACTATGTTATTAACA
GACCGAAACTTAAATACATCTTTCTTTGATCCAATCGGAGGAGGAGATCCTATTTTATAC
CAACACTTATTCTGATTCTTTGGACACCCAGAAGTTTATATTTTAATTTTACCCGGATTT
GGAATAATTTCTCATATTATTACTCAAGAAAGTGGAAAAAAGGAAACATTTGGAACCTTA
GGAATAATTTATGCTATATTAACAATTGGATTATTGGGATTTATTGTTTGAGCTCATCAT
ATATTTACAGTAGGTATAGACGTAGATACTCGAGCTTATTTTACTTCAGCAACTATAATT
ATTGCTGTTCCTACAGGAATTAAAATTTTTAGTTGATTAGCAACTTTACACGGAACTCAA
TTAACATATAGTCAG
>MLB_Ae.aegpti_32
AACATTAGGAGCCCCTGATATAGCTTTTCCTCGAATAAATAATATAAGTTTTTGAATACT
ACCTCCTTCATTGACTCTTCTATTATCAAGCTCAATAGTAGAAAATGGGGCAGGAACTGG
GTGAACAGTTTATCCTCCTCTCTCTTCAGGAACAGCTCATGCTGGAGCTTCTGTTGATTT
AGCTATTTTTTCTCTTCATTTAGCTGGAATTTCCTCAATTTTAGGGGCAGTAAATTTTAT
TACAACTGTAATTAATATACGATCGTCAGGAATTACTTTAGATCGACTACCCTTATTTGT
TTGATCTGTAGTTATTACAGCTATCTTATTACTTCTTTCTCTTCCTGTTTTAGCTGGAGC
TATTACTATGTTATTAACAGACCGAAACTTAAATACATCTTTCTTTGATCCAATCGGAGG
AGGAGATCCTATTTTATACCAACACTTATTCTGATTCTTTGGACACCCAGAAGTTTATAT
TTTAATTTTACCCGGATTTGGAATAATTTCTCATATTATTACTCAAGAAAGTGGAAAAAA
GGAAACATTTGGAACTTTAGGAATAATTTATGCTATATTAACAATTGGATTATTGGGATT
TATTGTTTGAGCTCATCATATATTTACAGTAGGTATAGACGTAGATACTCGAGCTTATTT
TACTTCAGCAACTATAATTATTGCTGTTCCTACAGGAATTAAAATTTTTAGTTGATTAGG
AACTTTACACGGAACTCAATTAACATATAGTCCGACCCTCCCCATGATAAAACAGAATGA
TAAGGCGGAGGTGGGACAGGCATGCGTGGGTCAATTTACAACCTAATGATACGGAAGGCT
GGACCAGGGGGGAGGGGTAAAATTACATGCGAA
>KSM_Ae.aegpti_01
TATAATTCGGAGGATTATTGAGAAATTAGATTAGTTCCTTTAATATTAGGAGCCCCTGAT
ATAGCCTTTCCTCGAATAAATAATATAAGTTTTTGAATACTACCTCCTTCATTGACTCTT
CTATTATCAAGCTCAATAGTAGAAAATGGAGCAGGAACTGGGTGAACAGTTTATCCTCCT

CTCTCTTCAGGAACAGCTCATGCTGGAGCTTCTGTTGATTTAGCTATTTTTTCTCTTCAT
TTAGCTGGAATTTCCTCAATTTTAGGGGCAGTAAATTTTATTACAACTGTAATTAATATA
CGATCGTCAGGAATTACTTTAGATCGACTACCCTTATTTGTTTGATCTGTAGTTATTACA
GCTATCTTATTACTTCTTTCTCTTCCTGTTTTAGCTGGAGCTATTACTATGTTATTAACA
GACCGAAACTTAAATACATCTTTCTTTGATCCAATCGGAGGAGGAGATCCTATTTTATAC
CAACACTTATTCTGATTCTTTGGACACCCAGAAGTTTATATTTTAATTTTACCCGGATTT
GGAATAATTTCTCATATTATTACTCAAGAAAGTGGAAAAAAGGAAACATTTGGAACTTTA
GGAATAATTTATGCTATATTAACAATTGGATTATTGGGGTTTATTGTTTGAGCTCGTCTT
ATATTTACAGTACGTATAGACTTAGATGCTAGGGGTGATTTTAGTGGTCCTACTAAAATG
AAGCTGTTCCTATCGGAAATAAACATATATTGATTGTATATCTGCACGAAACTCTATATA
GATATAATAAATCCCTCCACAAAAAAA
>KSM_Ae.aegpti_02
TTTATAGTATGCCATTATAATTGGAGGATTTGGAAATTGATTAGTTCCTTTAATATTAGG
AGCCCCTGATATAGCTTTCCCTCGAATGAATAATATAAGTTTTTGAATACTACCTCCTTC
ATTGACTCTTCTATTATCAAGCTCAATAGTAGAAAATGGAGCAGGAACTGGGTGAACAGT
TTATCCTCCTCTCTCTTCAGGAACAGCTCATGCTGGAGCTTCTGTTGATTTAGCTATTTT
TTCTCTTCATTTAGCTGGAATTTCCTCAATTTTAGGGGCAGTAAATTTTATTACAACTGT
AATTAATATACGATCGTCAGGGATTACTTTAGATCGACTACCCTTATTTGTTTGATCTGT
AGTTATTACAGCTATCTTATTACTTCTTTCTCTTCCTGTTTTAGCTGGAGCTATTACTAT
ATTATTAACAGACCGAAACTTAAATACATCTTTCTTTGATCCAATCGGAGGGGGAGACCC
TATTTTATACCAACACTTATTTTGATTCTTTGGGCACCCAGAAGTTTATATTTTAATTTT
ACCCGGATTTGGAATAATTTCTCATATTATTACTCAAGAAAGTGGAAAAAAGGAAACATT
TGGAACTTTAGGAATAATTTATGCTATATTAACAATTGGATTATTGGGATTTATTGTTTG
AGCTCATCATATATTTACAGTAGGTATAGATGTAGATACTCGAGCTTATTTTACTTCAGC
AACTATAATTATTGCTGTTCCTACAGGAATTAAAATTTTTAGTTGATTAGCAACTTTACA
TGGAAATCAATTAACATATAGTCCACTCCCCCCCAAAAAAAAAAAAAAAAAAAAAAAAAA
A
>KSM_Ae.aegpti_03
TCATTGCGAGTGTATTTGGAAATTAGATTAGTTCCTTTAATATTAGGAGCCCCTGATATA
GCTTTTCCTCGAATAAATAATATAAGTTTTTGAATACTACCTCCTTCATTGACTCTTCTA
TTATCAAGCTCAATAGTAGAAAATGGGGCAGGAACTGGGTGAACAGTTTATCCTCCTCTC
TCTTCAGGAACAGCTCATGCTGGAGCTTCTGTTGATTTAGCTATTTTTTCTCTTCATTTA
GCTGGAATTTCCTCAATTTTAGGGGCAGTAAATTTTATTACAACTGTAATTAATATACGA
TCGTCAGGAATTACTTTAGATCGACTACCCTTATTTGTTTGATCTGTAGTTATTACAGCT
ATCTTATTACTTCTTTCTCTTCCTGTTTTAGCTGGAGCTATTACTATGTTATTAACAGAC
CGAAACTTAAATACATCTTTCTTTGATCCAATCGGAGGAGGAGATCCTATTTTATACCAA
CACTTATTCTGATTCTTTGGACACCCCGAAGTTTATATTTTAATTTTACCCGGATTTGGA
ATAATTTCTCATATTATTACTCAAGAAAGTGGAAAAAAGGAAACATTTGGAACTTTAGGA
ATAATTTATGCTATATTAACAATTGGATTATTGGGATTTATTGTTTGAGCTCATCCTATA
TTTACAGTACGTGTAGACCTTAATACTCCAGCTTATTTTTACTTCCAACAATAATAATAA
GCGCTGTCCCTACCGAATTAAAATATTTATAGTTGATTGACACTTTCACAGAGAACAAAT
TAACATAAGGCCCCCCCCCAACAAAAAAAAAAAAAAAAAAAAAAAAAA
>KSM_Ae.aegpti_04
TATAATTGGAGGATTTGGAAATTGATTAGTTCCTTTAATATTAGGAGCCCCTGATATAGC
TTTCCCTCGAATGAATAATATAAGTTTTTGAATACTACCTCCTTCATTGACTCTTCTATT
ATCAAGCTCAATAGTAGAAAATGGAGCAGGAACTGGGTGAACAGTTTATCCTCCTCTCTC
TTCAGGAACAGCTCATGCTGGAGCTTCTGTTGATTTAGCTATTTTTTCTCTTCATTTAGC
TGGAATTTCCTCAATTTTAGGGGCAGTAAATTTTATTACAACTGTAATTAATATACGATC
GTCAGGGATTACTTTAGATCGACTACCCTTATTTGTTTGATCTGTAGTTATTACAGCTAT
CTTATTACTTCTTTCTCTTCCTGTTTTAGCTGGAGCTATTACTATATTATTAACAGACCG

AAACTTAAATACATCTTTCTTTGATCCAATCGGAGGGGGAGACCCTATTTTATACCAACA
CTTATTTTGATTCTTTGGACACCCAGAAGTTTATATTTTAATTTTACCCGGATTTGGGAT
AATTTCTCATATTATTACTCAAGAAAGTGGAAAAAAGGAAACATTTGGAACTTTAGGAAT
AATTTATGCTATATTAACAATTGGATTATTGGGATTTATTGTTTGAGCTCATCATATATT
TACAGTAGGTATAGATGTAGATACTCGAGCTTATTTTACTTCAGCAACTATAATTATTGC
TGTTCCTACAGGAATTAAAATTTTTAGTTGATTAGCAACTTTACACGGAACTCAATTAAC
ATATAGTCCAGCCGTCCCAATTATA
>KSM_Ae.aegpti_05
ATTTAGAGGATTTGGAAATTGATTAGTTCCTTTAATATTAGGAGCCCCTGATATAGCTTT
TCCTCGAATAAATAATATAAGTTTTTGAATACTACCTCCTTCATTGACTCTTCTATTATC
AAGCTCAATAGTAGAAAATGGGGCAGGAACTGGGTGAACAGTTTATCCTCCTCTCTCTTC
AGGAACAGCTCATGCTGGAGCTTCTGTTGATTTAGCTATTTTTTCTCTTCATTTAGCTGG
AATTTCCTCAATTTTAGGGGCAGTAAATTTTATTACAACTGTAATTAATATACGATCGTC
AGGAATTACTTTAGATCGACTACCCTTATTTGTTTGATCTGTAGTTATTACAGCTATCTT
ATTACTTCTTTCTCTTCCTGTTTTAGCTGGAGCTATTACTATGTTATTAACAGACCGAAA
CTTAAATACATCTTTCTTTGATCCAATCGGAGGAGGAGATCCTATTTTATACCAACACTT
ATTCTGATTCTTTGGACACCCAGAAGTTTATATTTTAATTTTACCCGGATTTGGAATAAT
TTCTCATATTATTACTCAAGAAAGTGGAAAAAAGGAAACATTTGGAACTTTAGGAATAAT
TTATGCTATATTAACAATTGGATTATTGGGATTTATTGTTTGAGCTCATCATATATTTAC
AGTAGGTATAGACGTAGATACTCGAGCTTATTTTACTTCAGCAACTATAATTATTGCTGT
TCCTACAGGAATTAAAATTTTTAGTTGATTAGCAACTTTACACGGAACTCAATTAACATA
TAGTCAGCTCTCTTTAAAAAAAAAAAAAAAAAAAAAAAAA
>KSM_Ae.aegpti_06
TATAATATGAGAGGATTTGAGAAATTGATTAGTTCCTTTAATATTAGGAGCCCCTGATAT
AGCTTTCCCTCGAATGAATAATATAAGTTTTTGAATACTACCTCCTTCATTGACTCTTCT
ATTATCAAGCTCAATAGTAGAAAATGGAGCAGGAACTGGGTGAACAGTTTATCCTCCTCT
CTCTTCAGGAACAGCTCATGCTGGAGCTTCTGTTGATTTAGCTATTTTTTCTCTTCATTT
AGCTGGAATTTCCTCAATTTTAGGGGCAGTAAATTTTATTACAACTGTAATTAATATACG
ATCGTCAGGGATTACTTTAGATCGACTACCCTTATTTGTTTGATCTGTAGTTATTACAGC
TATCTTATTACTTCTTTCTCTTCCTGTTTTAGCTGGAGCTATTACTATATTATTAACAGA
CCGAAACTTAAATACATCTTTCTTTGATCCAATCGGAGGGGGAGACCCTATTTTATACCA
ACACTTATTTTGATTCTTTGGACACCCAGAAGTTTATATTTTAATTTTACCCGGATTTGG
GATAATTTCTCATATTATTACTCAAGAAAGTGGAAAAAAGGAAACATTTGGAACTTTAGG
AATAATTTATGCTATATTAACAATTGGATTATTGGGATTTATTGTTTGAGCTCATCATAT
ATTTACAGTAGGTATAGATGTAGATACTCGAGCTTATTTTACTTCAGCAACTATAATTAT
TGCTGTTCCTACAGGAATTAAAATTTTTAGTTGATTAGCAACTTTACACGGAACTCAATT
AACATATAGTCCAGCCTCTT
>KSM_Ae.aegpti_08
ATACCAATTATAATTGGAGGATTTGGAAATTGATTAGTTCCTTTAATATTAGGAGCCCCT
GATATAGCCTTTCCTCGAATAAATAATATAAGTTTTTGAATACTACCTCCTTCATTGACT
CTTCTATTATCAAGCTCAATAGTAGAAAATGGGGCAGGAACTGGGTGAACAGTTTATCCT
CCTCTCTCTTCAGGAACAGCTCATGCTGGAGCTTCTGTTGATTTAGCTATTTTTTCTCTT
CATTTAGCTGGAATTTCCTCAATTTTAGGGGCAGTAAATTTTATTACAACTGTAATTAAT
ATACGATCATCAGGAATTACTTTAGATCGACTACCCTTATTTGTTTGATCTGTAGTTATT
ACAGCTATCTTATTACTTCTTTCTCTTCCTGTTTTAGCTGGAGCTATTACTATATTATTA
ACAGACCGAAACTTAAATACATCTTTCTTTGACCCAATCGGAGGAGGAGATCCTATTTTA
TACCAACACTTATTCTGATTCTTTGGACACCCAGAAGTTTATATTTTAATTTTACCCGGA
TTTGGAATAATTTCTCATATTATTACTCAAGAAAGTGGAAAAAAGGAAACATTTGGAACT
TTAGGAATAATTTATGCTATATTAACAATTGGATTATTGGGATTTATTGTTTGAGCTCAT
CATATATTTACAGTAGGTATAGACGTAGATACTCGAGCTTATTTTACTTCAGCAACTATA

ATTATTGCTGTTCCTACAGGAATTAAAATTTTTAGTTGATTAGCAACTTTACACGGAACT
CAATTAACATATAGTCCAGCCTCCTTTTTAATAAATAAAAAAAAAAAAAAAA
>KSM_Ae.aegpti_09
GCGCAGGACTATATAGAGTAAATATAGATTAGTTCCTTTAATATTAGGAGCCCCTGATAT
AGCCTTTCCTCGAATAAATAATATAAGTTTTTGAATACTACCTCCTTCATTGACTCTTCT
ATTATCAAGCTCAATAGTAGAAAATGGGGCAGGAACTGGGTGAACAGTTTATCCTCCTCT
CTCTTCAGGAACAGCTCATGCTGGAGCTTCTGTTGATTTAGCTATTTTTTCTCTTCATTT
AGCTGGAATTTCCTCAATTTTAGGGGCAGTAAATTTTATTACAACTGTAATTAATATACG
ATCGTCAGGAATTACTTTAGATCGACTACCCTTATTTGTTTGATCTGTAGTTATTACAGC
TATCTTATTACTTCTTTCTCTTCCTGTTTTAGCTGGAGCTATTACTATGTTATTAACAGA
CCGAAACTTAAATACATCTTTCTTTGATCCAATCGGAGGAGGAGATCCTATTTTATACCA
ACACTTATTCTGATTCTTTGGACACCCAGAAGTTTATATTTTAATTTTACCCGGATTTGG
AATAATTTCTCATATTATTACTCAAGAAAGTGGAAAAAAGGAAACATTTGGAACTTTAGG
AATAATTTATGCTATATTAACAATTGGATTATTGGGATTTATTGTTTGAGCTCATCATAT
ATTTACAGTAGGTATAGACGTAGATACTCGAGCTTATTTTACTTCAGCAACTATAATTAT
TGCTGTTCCTACAGGAATTAAAATTTTTAGTTGATTAGCAACTTTACACGGAACTCAATT
AACATATAGTCCAGCCTCT
>KSM_Ae.aegpti_11
ATTATAATTGAGAGGATTTGGAAATAAGATTAGTTCCTTTAATATTAGGAGCCCCTGATA
TAGCCTTTCCTCGAATAAATAATATAAGTTTTTGAATACTACCTCCTTCATTGACTCTTC
TATTATCAAGCTCAATAGTAGAAAATGGAGCAGGAACTGGATGAACAGTTTATCCTCCTC
TCTCTTCAGGAACAGCTCATGCTGGAGCTTCTGTTGATTTAGCTATTTTTTCTCTTCATT
TAGCTGGAATTTCCTCAATTTTAGGGGCAGTAAATTTTATTACAACTGTAATTAATATAC
GATCGTCAGGAATTACTTTAGATCGACTACCCTTATTTGTTTGATCTGTAGTTATTACAG
CTATCTTATTACTTCTTTCTCTTCCTGTTTTAGCTGGAGCTATTACTATATTATTAACAG
ACCGAAACTTAAATACATCTTTCTTTGACCCAATCGGAGGAGGAGATCCTATTTTATATC
AACACTTATTCTGATTCTTTGGACACCCAGAAGTTTATATTTTAATTTTACCCGGATTTG
GAATAATTTCTCATATTATTACTCAAGAAAGTGGAAAAAAGGAAACATTCGGAACTTTAG
GAATAATTTATGCTATATTAACAATTGGATTATTGGGATTTATTGTTTGAGCTCATCATA
TATTTACAGTAGGTATAGACGTAGATACTCGAGCTTATTTTACTTCAGCAACTATAATTA
TTGCTGTTCCTACAGGAATTAAAATTTTTAGTTGATTAGCAACTTTACACGGAACTCAAT
TAACATATAGTCCAGCTCCTTCAGAAAAAAAAAAAAAAAAAAAAAA
>KSM_Ae.aegpti_12
ATTATAATTAGGAGGATTTAGGAAATTGATTAGTTCCTTTAATATTAGGAGCCCCTGATA
TAGCCTTTCCTCGAATAAATAATATAAGTTTTTGAATACTACCTCCTTCATTGACTCTTC
TATTATCAAGCTCAATAGTAGAAAATGGAGCAGGAACTGGATGAACAGTTTATCCTCCTC
TCTCTTCAGGAACAGCTCATGCTGGAGCTTCTGTTGATTTAGCTATTTTTTCTCTTCATT
TAGCTGGAATTTCCTCAATTTTAGGGGCAGTAAATTTTATTACAACTGTAATTAATATAC
GATCGTCAGGAATTACTTTAGATCGACTACCCTTATTTGTTTGATCTGTAGTTATTACAG
CTATCTTATTACTTCTTTCTCTTCCTGTTTTAGCTGGAGCTATTACTATATTATTAACAG
ACCGAAACTTAAATACATCTTTCTTTGACCCAATCGGAGGAGGAGATCCTATTTTATATC
AACACTTATTCTGATTCTTTGGACACCCAGAAGTTTATATTTTAATTTTACCCGGATTTG
GAATAATTTCTCATATTATTACTCAAGAAAGTGGAAAAAAGGAAACATTCGGAACTTTAG
GAATAATTTATGCTATATTAACAATTGGATTATTGGGATTTATTGTTTGAGCTCATCATA
TATTTACAGTAGGTATAGACGTAGATACTCGAGCTTATTTTACTTCAGCAACTATAATTA
TTGCTGTTCCTACAGGAATTAAAATTTTTAGTTGATTAGCAACTTTACACGGAACTCAAT
TAACATATAGTCCAGCCTCTTCTTTAAAAAATAAAAAAAAA
>KSM_Ae.aegpti_13
CCAATTATAATTCGCGAGGATTTGGGAATATTGATTAGTTCCTTTAATATTAGGAGCCCC
TGATATAGCCTTTCCTCGAATAAATAATATAAGTTTTTGAATACTACCTCCTTCATTGAC

TCTTCTATTATCAAGCTCAATAGTAGAAAATGGGGCAGGAACTGGGTGAACAGTTTATCC
TCCTCTCTCTTCAGGAACAGCTCATGCTGGAGCTTCTGTTGATTTAGCTATTTTTTCTCT
TCATTTAGCTGGAATTTCCTCAATTTTAGGGGCAGTAAATTTTATTACAACTGTAATTAA
TATACGATCGTCAGGAATTACTTTAGATCGACTACCCTTATTTGTTTGATCTGTAGTTAT
TACAGCTATCTTATTACTTCTTTCTCTTCCTGTTTTAGCTGGAGCTATTACTATGTTATT
AACAGACCGAAACTTAAATACATCTTTCTTTGATCCAATCGGAGGAGGAGATCCTATTTT
ATACCAACACTTATTCTGATTCTTTGGACACCCAGAAGTTTATATTTTAATTTTACCCGG
ATTTGGAATAATTTCTCATATTATTACTCAAGAAAGTGGAAAAAAGGAAACATTTGGAAC
TTTAGGAATAATTTATGCTATATTAACAATTGGATTATTGGGATTTATTGTTTGAGCTCA
TCATATATTTACAGTAGGTATAGACGTAGATACTCGAGCTTATTTTACTTCAGCAACTAT
AATTATTGCTGTTCCTACAGGAATTAAAATTTTTAGTTGATTAGCAACTTTACACGGAAC
TCAATTAACATATAGTCCAGCCCTCCTCTGA
>KSM_Ae.aegpti_14
ACTCACTATAAAATAATAATAACCGGTTCTTGAGGAGAGCCCCCGAAGATCTTTTCCTCG
AAAGAAAAAAATAAATTTTTTAAACCCCCTCCCTCCATGACTCTTTTATTTATTGCTCAA
ATAATAAAAATGGGGGGGAGAGGGTGGGAGTTTTCCCTCCTCCTTTCAAGGAAGAAGCAG
GCGGAACCTTCGGTGAATTTATTTTTTTTTTTTTTTCCTTTTATTTGGATTTTCCTTGTTT
TTGGGGGGGTAAATAACTTATTACACTAATGAATATACGATGGAGGGAATTACTTTACAT
CGACTACCCTGTGTTGTTTGATGTGATGTTATTACAGCATTTTATTACTTCTTTTCTTTT
TGTTTGTGTTTGGGTCTTAATCTAAGTGATTCCCGAACGAAATCTAAATTCCTCTTGTTC
TATATCCGAGGCGAAGGGGGAAGAAGATAT
>KSM_Ae.aegpti_15
ATACCAATTATAATTCGTAGAGGATTTTGAGAAATTGATTAGTTCCTTTAATATTAGGAG
CCCCTGATATAGCCTTTCCTCGAATAAATAATATAAGTTTTTGAATACTACCTCCTTCAT
TGACTCTTCTATTATCAAGCTCAATAGTAGAAAATGGGGCAGGAACTGGGTGAACAGTTT
ATCCTCCTCTCTCTTCAGGAACAGCTCATGCTGGAGCTTCTGTTGATTTAGCTATTTTTT
CTCTTCATTTAGCTGGAATTTCCTCAATTTTAGGGGCAGTAAATTTTATTACAACTGTAA
TTAATATACGATCGTCAGGAATTACTTTAGATCGACTACCCTTATTTGTTTGATCTGTAG
TTATTACAGCTATCTTATTACTTCTTTCTCTTCCTGTTTTAGCTGGAGCTATTACTATGT
TATTAACAGACCGAAACTTAAATACATCTTTCTTTGATCCAATCGGAGGAGGAGATCCTA
TTTTATACCAACACTTATTCTGATTCTTTGGACACCCAGAAGTTTATATTTTAATTTTAC
CCGGATTTGGAATAATTTCTCATATTATTACTCAAGAAAGTGGAAAAAAGGAAACATTTG
GAACTTTAGGAATAATTTATGCTATATTAACAATTGGATTATTGGGATTTATTGTTTGAG
CTCATCATATATTTACAGTAGGTATAGACGTAGATACTCGAGCTTATTTTACTTCAGCAA
CTATAATTATTGCTGTTCCTACAGGAATTAAAATTTTTAGTTGATTAGCAACTTTACACG
GAACTCAATTAACATATAGTCCAGCTCCTTCTAAAAAAAAAAAAAAA
>KSM_Ae.aegpti_18
CCATTATAATTTAGGAGGATTTGGAAATTTCGATTAGTTCCTTTAATATTAGGAGCCCCT
GATATAGCTTTTCCTCGAATAAATAATATAAGTTTTTGAATACTACCTCCTTCATTGACT
CTTCTATTATCAAGCTCAATAGTAGAAAATGGGGCAGGAACTGGGTGAACAGTTTATCCT
CCTCTCTCTTCAGGAACAGCTCATGCTGGAGCTTCTGTTGATTTAGCTATTTTTTCTCTT
CATTTAGCTGGAATTTCCTCAATTTTAGGGGCAGTAAATTTTATTACAACTGTAATTAAT
ATACGATCGTCAGGAATTACTTTAGATCGACTACCCTTATTTGTTTGATCTGTAGTTATT
ACAGCTATCTTATTACTTCTTTCTCTTCCTGTTTTAGCTGGAGCTATTACTATGTTATTA
ACAGACCGAAACTTAAATACATCTTTCTTTGATCCAATCGGAGGAGGAGATCCTATTTTA
TACCAACACTTATTCTGATTCTTTGGACACCCAGAAGTTTATATTTTAATTTTACCCGGA
TTTGGAATAATTTCTCATATTATTACTCAAGAAAGTGGAAAAAAGGAAACATTTGGAACT
TTAGGAATAATTTATGCTATATTAACAATTGGATTATTGGGATTTATTGTTTGAGCTCAT
CATATATTTACAGTAGGTATAGACGTAGATACTCGAGCTTATTTTACTTCAGCAACTATA
ATTATTGCTGTTCCTACAGGAATTAAGATTTTTAGTTGATTAGCAACTTTACACGGAACT

CAATTAACATATAGTCCGCTCCCCTCCAAAAAAAAAAAAAAAAAAAAAA
>KSM_Ae.aegpti_19
ATCGCCATTATAATTGAGAGGATTTGGAAATTAGATTAGTTCCTTTAATATTAGGAGCCC
CTGATATAGCTTTCCCTCGAATGAATAATATAAGTTTTTGAATACTACCTCCTTCATTGA
CTCTTCTATTATCAAGCTCAATAGTAGAAAATGGAGCAGGAACTGGGTGAACAGTTTATC
CTCCTCTCTCTTCAGGAACAGCTCATGCTGGAGCTTCTGTTGATTTAGCTATTTTTTCTC
TTCATTTAGCTGGAATTTCCTCAATTTTAGGGGCAGTAAATTTTATTACAACTGTAATTA
ATATACGATCGTCAGGGATTACTTTAGATCGACTACCCTTATTTGTTTGATCTGTAGTTA
TTACAGCTATCTTATTACTTCTTTCTCTTCCTGTTTTAGCTGGAGCTATTACTATATTAT
TAACAGACCGAAACTTAAATACATCTTTCTTTGATCCAATCGGAGGGGGAGACCCTATTT
TATACCAACACTTATTTTGATTCTTTGGGCACCCAGAAGTTTATATTTTAATTTTACCCG
GATTTGGAATAATTTCTCATATTATTACTCAAGAAAGTGGAAAAAAGGAAACATTTGGAA
CTTTAGGAATAATTTATGCTATATTAACAATTGGATTATTGGGATTTATTGTTTGAGCTC
ATCATATATTTACAGTAGGTATAGATGTAGATACTCGAGCTTATTTTACTTCAGCAACTA
TAATTATTGCTGTTCCTACCGGAATTAAAATTTTTAGTTGATTAGCAACTTTACATGGAA
CTCAATTAACATATAGTCAGCCCCAACAAAAAAATAAAAAAAAAAAAAAAAAA
>KSM_Ae.aegpti_20
GCCGAGGATTTAGTGTAATATTAGATTAGTTCCTTTAATATTAGGAGCCCCTGATATAGC
TTTCCCTCGAATGAATAATATAAGTTTTTGAATACTACCTCCTTCATTGACTCTTCTATT
ATCAAGCTCAATAGTAGAAAATGGAGCAGGAACTGGGTGAACAGTTTATCCTCCTCTCTC
TTCAGGAACAGCTCATGCTGGAGCTTCTGTTGATTTAGCTATTTTTTCTCTTCATTTAGC
TGGAATTTCCTCAATTTTAGGGGCAGTAAATTTTATTACAACTGTAATTAATATACGATC
GTCAGGGATTACTTTAGATCGACTACCCTTATTTGTTTGATCTGTAGTTATTACAGCTAT
CTTATTACTTCTTTCTCTTCCTGTTTTAGCTGGAGCTATTACTATATTATTAACAGACCG
AAACTTAAATACATCTTTCTTTGATCCAATCGGAGGGGGAGACCCTATTTTATACCAACA
CTTATTTTGATTCTTTGGACACCCAGAAGTTTATATTTTAATTTTACCCGGATTTGGGAT
AATTTCTCATATTATTACTCAAGAAAGTGGAAAAAAGGAAACATTTGGAACTTTAGGAAT
AATTTATGCTATATTAACAATTGGATTATTGGGATTTATTGTTTGAGCTCATCATATATT
TACAGTAGGTATAGATGTAGATACTCGAGCTTATTTTACTTCAGCAACTATAATTATTGC
TGTTCCTACAGGAATTAAAATTTTTAGTTGATTAGCAACTTTACACGGAACTCAATTAAC
ATATAGTCCAGCCTATTTATTAATTACAAAAAAAAAAATAAAAAAG
>KSM_Ae.aegpti_21
ATTATATATATGAGAGGATTTGGAAATTAGATTAGTTCCTTTAATATTAGGAGCCCCTGA
TATAGCTTTTCCTCGAATAAATAATATAAGTTTTTGAATACTACCTCCTTCATTGACTCT
TCTATTATCAAGCTCAATAGTAGAAAATGGGGCAGGAACTGGGTGAACAGTTTATCCTCC
TCTCTCTTCAGGAACAGCTCATGCTGGAGCTTCTGTTGATTTAGCTATTTTTTCTCTTCA
TTTAGCTGGAATTTCCTCAATTTTAGGGGCAGTAAATTTTATTACAACTGTAATTAATAT
ACGATCGTCAGGAATTACTTTAGATCGACTACCCTTATTTGTTTGATCTGTAGTTATTAC
AGCTATCTTATTACTTCTTTCTCTTCCTGTTTTAGCTGGAGCTATTACTATGTTATTAAC
AGACCGAAACTTAAATACATCTTTCTTTGATCCAATCGGAGGAGGAGACCCTATTTTATA
CCAACACTTATTCTGATTCTTTGGACACCCAGAAGTTTATATTTTAATTTTACCCGGATT
TGGAATAATTTCTCATATTATTACTCAAGAAAGTGGAAAAAAGGAAACATTTGGAACTTT
AGGAATAATTTATGCTATATTAACAATTGGATTATTGGGATTTATTGTTTGAGCTCATCA
TATATTTACAGTAGGTATAGACGTAGATACTCGAGCTTATTTTACTTCAGCAACTATAAT
TATTGCTGTTCCTACAGGAATTAAAATTTTTAGTTGATTAGCAACTTTACACGGAACTCA
ATTAACATATAGTCCGCCTCTCCCTAATAAAAAAAAAAAAAAAAAA
>KSM_Ae.aegpti_22
TACCAATTATAATTGGAGGATTTGGAAATTGATTAGTTCCTTTAATATTAGGAGCCCCTG
ATATAGCCTTTCCTCGAATAAATAATATAAGTTTTTGAATACTACCTCCTTCATTGACTC
TTCTATTATCAAGCTCAATAGTAGAAAATGGAGCAGGAACTGGGTGAACAGTTTATCCTC

CTCTCTCTTCAGGAACAGCTCATGCTGGAGCTTCTGTTGATTTAGCTATTTTTTCTCTTC
ATTTAGCTGGAATTTCCTCAATTTTAGGGGCAGTAAATTTTATTACAACTGTAATTAATA
TACGATCGTCAGGAATTACTTTAGATCGACTACCCTTATTTGTTTGATCTGTAGTTATTA
CAGCTATCTTATTACTTCTTTCTCTTCCTGTTTTAGCTGGAGCTATTACTATGTTATTAA
CAGACCGAAACTTAAATACATCTTTCTTTGATCCAATCGGAGGAGGAGATCCTATTTTAT
ACCAACACTTATTCTGATTCTTTGGACACCCAGAAGTTTATATTTTAATTTTACCCGGAT
TTGGAATAATTTCTCATATTATTACTCAAGAAAGTGGAAAAAAGGAAACATTTGGAACTT
TAGGAATAATTTATGCTATATTAACAATTGGATTATTGGGATTTATTGTTTGAGCTCATC
ATATATTTACAGTAGGTATAGACGTAGATACTCGAGCTTATTTTACTTCAGCAACTATAA
TTATTGCTGTTCCTACAGGAATTAAGATTTTTAGTTGATTAGCAACTTTACACGGAACTC
AATTAACATATAGTCCAGCCTCTTTTTAAAATAAAATAAAAAAAAA
>KSM_Ae.aegpti_23
AGGATTTTAGGATATTAGATTAGTTCCTTTAATATTAGGAGCCCCTGATATAGCTTTCCC
TCGAATGAATAATATAAGTTTTTGAATACTACCTCCTTCATTGACTCTTCTATTATCAAG
CTCAATAGTAGAAAATGGAGCAGGAACTGGGTGAACAGTTTATCCTCCTCTCTCTTCAGG
AACAGCTCATGCTGGAGCTTCTGTTGATTTAGCTATTTTTTCTCTTCATTTAGCTGGAAT
TTCCTCAATTTTAGGGGCAGTAAATTTTATTACAACTGTAATTAATATACGATCGTCAGG
GATTACTTTAGATCGACTACCCTTATTTGTTTGATCTGTAGTTATTACAGCTATCTTATT
ACTTCTTTCTCTTCCTGTTTTAGCTGGAGCTATTACTATATTATTAACAGACCGAAACTT
AAATACATCTTTCTTTGATCCAATCGGAGGGGGAGACCCTATTTTATACCAACACTTATT
TTGATTCTTTGGGCACCCAGAAGTTTATATTTTAATTTTACCCGGATTTGGAATAATTTC
TCATATTATTACTCAAGAAAGTGGAAAAAAGGAAACATTTGGAACTTTAGGAATAATTTA
TGCTATATTAACAATTGGATTATTGGGATTTATTGTTTGAGCTCATCATATATTTACAGT
AGGTATAGATGTAGATACTCGAGCTTATTTTACTTCAGCAACTATAATTATTGCTGTTCC
TACAGGAATTAAAATTTTTAGTTGATTAGCAACTTTACATGGAACTCAATTAACATATAG
TCCAGCTCTCCTTTAAATAACAAAAAAAAAAAAAAAAA
>KSM_Ae.aegpti_24
TAGACTAGTTCCTTTAATATTAGGAGCCCCTGATATAGCCTTTCCTCGAATAAATAATAT
AAGTTTTTGAATACTACCTCCTTCATTGACTCTTCTATTATCAAGCTCAATAGTAGAAAA
TGGAGCAGGAACTGGGTGAACAGTTTATCCTCCTCTCTCTTCAGGAACAGCTCATGCTGG
AGCTTCTGTTGATTTAGCTATTTTTTCTCTTCATTTAGCTGGAATTTCCTCAATTTTAGG
GGCAGTAAATTTTATTACAACTGTAATTAATATACGATCGTCAGGAATTACTTTAGATCG
ACTACCCTTATTTGTTTGATCTGTAGTTATTACAGCTATCTTATTACTTCTTTCTCTTCC
TGTTTTAGCTGGAGCTATTACTATGTTATTAACAGACCGAAACTTAAATACATCTTTCTT
TGATCCAATCGGAGGAGGAGATCCTATTTTATACCAACACTTATTCTGATTCTTTGGACA
CCCAGAAGTTTATATTTTAATTTTACCCGGATTTGGAATAATTTCTCATATTATTACTCA
AGAAAGTGGAAAAAAGGAAACATTTGGAACTTTAGGAATAATTTATGCTATATTAACAAT
TGGATTATTGGGATTTATTGTTTGAGCTCATCATATATTTACAGTAGGTATAGACGTAGA
TACTCGAGCTTATTTTACTTCAGCAACTATAATTATTGCTGTTCCTACAGGAATTAAAAT
TTTTAATTGATTAGCAACTTTACACGGAACTCAATTAACATATAGTCCAGCCCTCCCAAT
GAACAAAAAA
>KSM_Ae.aegpti_25
GTAACAGCTCAGGCATGGCCCAGCCCTTCTTGATCATTAGGCTGAGATTGTAACATCTCA
GGCATGGCCCCGCCCTTCTTTTCTTTTTACACTACTTTTTTAATCTCTCATGCCCTCCCC
CCCCTTTTTTATATAAGAGGGGAGGAGAGAGGGGGGTCAAGAACCCCCCACTCTTTACAGA
AAGAGGGGGGGTAGAGCAACAGCGGTGAACAATTTTCTCAAATCATTAGGCGGGGATTTC
CTCTAAATGATAGGCACAAATTCATTACAACGGTGTAAATTCCTACCAAAGAAATCCTTC
AAACCCCAAGCCATATAGCTTGGGCTGTGGCAACAACAGTATTACTGCTATTTCATCCCC
TCCCGG
>KSM_Ae.aegpti_26

CCAATTATAATTGGAGGTATTTGGAAATTGATTAGTTCCTTTAATATTAGGAGCCCCTGA
TATAGCCTTTCCTCGAATAAATAATATAAGTTTTTGAATACTACCTCCTTCATTGACTCT
TCTATTATCAAGCTCAATAGTAGAAAATGGGGCAGGAACTGGGTGAACAGTTTATCCTCC
TCTCTCTTCAGGAACAGCTCATGCTGGAGCTTCTGTTGATTTAGCTATTTTTTCTCTTCA
TTTAGCTGGAATTTCCTCAATTTTAGGGGCAGTAAATTTTATTACAACTGTAATTAATAT
ACGATCGTCAGGAATTACTTTAGATCGACTACCCTTATTTGTTTGATCTGTAGTTATTAC
AGCTATCTTATTACTTCTTTCTCTTCCTGTTTTAGCTGGAGCTATTACTATGTTATTAAC
AGACCGAAACTTAAATACATCTTTCTTTGATCCAATCGGAGGAGGAGATCCTATTTTATA
CCAACACTTATTCTGATTCTTTGGACACCCAGAAGTTTATATTTTAATTTTACCCGGATT
TGGAATAATTTCTCATATTATTACTCAAGAAAGTGGAAAAAAGGAAACATTTGGAACTTT
AGGAATAATTTATGCTATATTAACAATTGGATTATTGGGATTTATTGTTTGAGCTCATCA
TATATTTACAGTAGGTATAGACGTAGATACTCGAGCTTATTTTACTTCAGCAACTATAAT
TATTGCTGTTCCTACAGGAATTAAAATTTTTAGTTGATTAGCAACTTTACACGGAACTCA
ATTAACATATAGTCCACCTCCCAAAAAAAAAAAAAAAAAAAAAAAAAAAA
>KSM_Ae.aegpti_27
ATACCAATTATAATTGGTGGATTTGAGAAATTGATTAGTTCCTTTAATATTAGGAGCCCC
TGATATAGCTTTCCCTCGAATGAATAATATAAGTTTTTGAATACTACCTCCTTCATTGAC
TCTTCTATTATCAAGCTCAATAGTAGAAAATGGAGCAGGAACTGGGTGAACAGTTTATCC
TCCTCTCTCTTCAGGAACAGCTCATGCTGGAGCTTCTGTTGATTTAGCTATTTTTTCTCT
TCATTTAGCTGGAATTTCCTCAATTTTAGGGGCAGTAAATTTTATTACAACTGTAATTAA
TATACGATCGTCAGGAATTACTTTAGATCGACTACCCTTATTTGTTTGATCTGTAGTTAT
TACAGCTATCTTATTACTTCTTTCTCTTCCTGTTTTAGCTGGAGCTATTACTATATTATT
AACAGACCGAAATTTAAATACATCTTTCTTTGACCCAATCGGAGGGGGAGACCCTATTTT
ATACCAACACTTATTTTGATTCTTTGGGCACCCAGAAGTTTATATTTTAATTTTACCCGG
ATTTGGGATAATTTCTCATATTATTACTCAAGAAAGTGGAAAAAAGGAAACATTTGGAAC
TTTAGGAATAATTTATGCTATATTAACAATTGGATTATTAGGATTTATTGTTTGAGCTCA
TCATATATTTACAGTAGGTATAGACGTAGATACTCGAGCTTATTTTACTTCAGCAACTAT
AATTATTGCTGTTCCTACAGGAATTAAAATTTTTAGTTGATTAGCAACTTTACACGGAAC
TCAATTAACATATAGTCAGCTCCCCTCAAAAAAAAAAAAAAAAAAAAAAAAAA
>KSM_Ae.aegpti_28
ATTATAATTGGAGGATTTGGAAATTAGATTAGTTCCTTTAATATTAGGAGCCCCTGATAT
AGCTTTCCCTCGAATAAATAATATAAGTTTTTGAATACTACCTCCTTCATTGACTCTTCT
ATTATCAAGCTCAATAGTAGAAAATGGGGCAGGAACTGGGTGAACAGTTTATCCTCCTCT
CTCTTCAGGAACAGCTCATGCTGGAGCTTCTGTTGATTTAGCTATTTTTTCTCTTCATTT
AGCTGGAATTTCCTCAATTTTAGGGGCAGTAAATTTTATTACAACTGTAATTAATATACG
ATCGTCAGGGATTACTTTAGATCGACTACCCTTATTTGTTTGATCTGTAGTTATTACAGC
TATCTTATTACTTCTTTCTCTTCCTGTTTTAGCTGGAGCTATTACTATATTATTAACAGA
CCGAAACTTAAATACATCTTTCTTTGATCCAATCGGAGGAGGAGACCCTATTTTATACCA
ACACTTATTTTGATTCTTTGGGCACCCAGAAGTTTATATTTTAATTTTACCCGGATTTGG
AATAATTTCTCATATTATTACTCAAGAAAGTGGAAAAAAGGAAACATTCGGAACTTTAGG
AATAATTTATGCTATATTAACAATTGGATTATTGGGATTTATTGTTTGAGCTCATCATAT
ATTTACAGTAGGTATAGACGTAGATACTCGAGCTTATTTTACTTCAGCAACTATAATTAT
TGCTGTTCCTACAGGAATTAAAATTTTTAGTTGATTAGCAACTTTACACGGAACTCAATT
AACATATAGTCCAGCCTCTCTCTTTAAACAAAAAAAAAAAAAAAA
>KSM_Ae.aegpti_29
TTATACCAATTATAATATGGAGGATTTGGAAATTGATTAGTTCCTTTAATATTAGGAGCC
CCTGATATAGCCTTTCCTCGAATAAATAATATAAGTTTTTGAATACTACCTCCTTCATTG
ACTCTTCTATTATCAAGCTCAATAGTAGAAAATGGAGCAGGAACTGGGTGAACAGTTTAT
CCTCCTCTCTCTTCAGGAACAGCTCATGCTGGAGCTTCTGTTGATTTAGCTATTTTTTCT
CTTCATTTAGCTGGAATTTCCTCAATTTTAGGGGCAGTAAATTTTATTACAACTGTAATT

AATATACGATCGTCAGGAATTACTTTAGATCGACTACCCTTATTTGTTTGATCTGTAGTT
ATTACAGCTATCTTATTACTTCTTTCTCTTCCTGTTTTAGCTGGAGCTATTACTATGTTA
TTAACAGACCGAAACTTAAATACATCTTTCTTTGATCCAATTGGAGGAGGAGATCCTATT
TTATACCAACACTTATTCTGATTCTTTGGACACCCAGAAGTTTATATTTTAATTTTACCC
GGATTTGGAATAATTTCTCATATTATTACTCAAGAAAGTGGAAAAAAGGAAACATTTGGA
ACTTTAGGAATAATTTATGCTATATTAACAATTGGATTATTGGGATTTATTGTTTGAGCT
CATCATATATTTACAGTAGGTATAGACGTAGATACTCGAGCTTATTTTACTTCAGCAACT
ATAATTATTGCTGTTCCTACAGGAATTAAAATTTTTAGTTGATTAGCAACTTTACACGGA
ACTCAATTAACATATAGTCCAGCCCTCTCTAAAAAAAAAAAAAAAAAAAAAAAAAA
>KSM_Ae.aegpti_30
ATTATAATTGGAGGATTTGGAAATTGATTAGTTCCTTTAATATTAGGAGCCCCTGATATA
GCTTTTCCTCGAATAAATAATATAAGTTTTTGAATACTACCTCCTTCATTGACTCTTCTA
TTATCAAGCTCAATAGTAGAAAATGGGGCAGGAACTGGGTGAACAGTTTATCCTCCTCTC
TCTTCAGGAACAGCTCATGCTGGAGCTTCTGTTGATTTAGCTATTTTTTCTCTTCATTTA
GCTGGAATTTCCTCAATTTTAGGGGCAGTAAATTTTATTACAACTGTAATTAATATACGA
TCGTCAGGAATTACTTTAGATCGACTACCCTTATTTGTTTGATCTGTAGTTATTACAGCT
ATCTTATTACTTCTTTCTCTTCCTGTTTTAGCTGGAGCTATTACTATGTTATTAACAGAC
CGAAACTTAAATACATCTTTCTTTGATCCAATCGGAGGAGGAGATCCTATTTTATACCAA
CACTTATTCTGATTCTTTGGACACCCAGAAGTTTATATTTTAATTTTACCCGGATTTGGA
ATAATTTCTCATATTATTACTCAAGAAAGTGGAAAAAAGGAAACATTTGGAACTTTAGGA
ATAATTTATGCTATATTAACAATTGGATTATTGGGATTTATTGTTTGAGCTCATCATATA
TTTACAGTAGGTATAGACGTAGATACTCGAGCTTATTTTACTTCAGCAACTATAATTATT
GCTGTTCCTACAGGAATTAAAATTTTTAGTTGATTAGCAACTTTACACGGAACTCAATTA
ACATATAGTCCGCTCCCCTCAAATAAATTTAAAAAAAAAAAAAAA
>KSM_Ae.aegpti_31
ACCAATTATAATTGGAGGATTATGGAAATTGATTAGTTCCTTTAATATTAGGAGCCCCTG
ATATAGCCTTTCCTCGAATAAATAATATAAGTTTTTGAATACTACCTCCTTCATTGACTC
TTCTATTATCAAGCTCAATAGTAGAAAATGGAGCAGGAACTGGGTGAACAGTTTATCCTC
CTCTCTCTTCAGGAACAGCTCATGCTGGAGCTTCTGTTGATTTAGCTATTTTTTCTCTTC
ATTTAGCTGGAATTTCCTCAATTTTAGGGGCAGTAAATTTTATTACAACTGTAATTAATA
TACGATCGTCAGGAATTACTTTAGATCGACTACCCTTATTTGTTTGATCTGTAGTTATTA
CAGCTATCTTATTACTTCTTTCTCTTCCTGTTTTAGCTGGAGCTATTACTATGTTATTAA
CAGACCGAAACTTAAATACATCTTTCTTTGATCCAATCGGAGGAGGAGATCCTATTTTAT
ACCAACACTTATTCTGATTCTTTGGACACCCAGAAGTTTATATTTTAATTTTACCCGGAT
TTGGAATAATTTCTCATATTATTACTCAAGAAAGTGGAAAAAAGGAAACATTTGGAACTT
TAGGAATAATTTATGCTATATTAACAATTGGATTATTGGGGTTTATTGTTTGAGCTCATC
ATATATTTACAGTAGGTATAGACGTAGATACTCGAGCTTATTTTACTTCAGCAACTATAA
TTATTGCTGTTCCTACAGGAATTAAAATTTTTAGTTGATTAGCAACTTTACACGGAACTC
AATTAACATATAGTCCAGCTCTCCTTAAAAAAAAAAAAAAAAAAAAAAAAA
>KSM_Ae.aegpti_32
AATACCAATTATAATTGGAGGATTTGGAAATTGATTAGTTCCTTTAATATTAGGAGCCCC
TGATATAGCTTTTCCTCGAATAAATAATATAAGTTTTTGAATACTACCTCCTTCATTGAC
TCTTCTATTATCAAGCTCAATAGTAGAAAATGGGGCAGGAACTGGGTGAACAGTTTATCC
TCCTCTCTCTTCAGGAACAGCTCATGCTGGAGCTTCTGTTGATTTAGCTATTTTTTCTCT
TCATTTAGCTGGAATTTCCTCAATTTTAGGGGCAGTAAATTTTATTACAACTGTAATTAA
TATACGATCGTCAGGAATTACTTTAGATCGACTACCCTTATTTGTTTGATCTGTAGTTAT
TACAGCTATCTTATTACTTCTTTCTCTTCCTGTTTTAGCTGGAGCTATTACTATGTTATT
AACAGACCGAAACTTAAATACATCTTTCTTTGATCCAATCGGAGGAGGAGATCCTATTTT
ATACCAACACTTATTCTGATTCTTTGGACACCCAGAAGTTTATATTTTAATTTTACCCGG
ATTTGGAATAATTTCTCATATTATTACTCAAGAAAGTGGAAAAAAGGAAACATTTGGAAC

TTTAGGAATAATTTATGCTATATTAACAATTGGATTATTGGGATTTATTGTTTGAGCTCA
TCATATATTTACAGTAGGTATAGACGTAGATACTCGAGCTTATTTTACTTCAGCAACTAT
AATTATTGCTGTTCCTACAGGAATTAAAATTTTTAGTTGATTAGCAACTTTACACGGAAC
TCAATTAACATATAGTCCAGCTTCTT
>KSM_Ae.aegpti_33
AGTATTGTAACCTCTCATAGCATGATTAGGCCCTTCTATGATCAATAGGCTACTCTGTTA
TACCCTTTCCCCGGAAAATTATATTTTTTTTTGAATAACACCTCCCTCTGTGACTCTTCT
ATTAACAAGCTCAATAATAAAAAAAGGAGGAAGAAGGGTGTGAACTGTTTATCCTCCTTC
TTCTTCAAGAACAGCTCATGGAGGAACTTCCGTTGATTAAACTATTTTTTTTTTTCATTT
AGCTGGGTTTCCTCCATTTTGGGGCAGTTTATTCTAATACGACTGTAATTAAAAAACGAT
CGGCAGGAATTACAACCGATCACCCCCCCTTGATTTGTGTGGGAGGTAATAATTAACTAA
ATACATCATATTCTTCCCGTTTTGGTGGGCTCGTGACCTTTTTATTTACAAACC
