# Supplementary material for: Genetic diversity of Aedes aegypti populations from Kisumu and Busia counties, western Kenya, and their vector competence for chikungunya virus
Source: PLoS One. 2025 Mar 25;20(3):e0289191. doi: 10.1371/journal.pone.0289191 (PMC11936183; doi:10.1371/journal.pone.0289191)
Supplement: S3 File — (DOCX) [file pone.0289191.s003.docx]

Supporting document

To determine the titer of the viruses used to infect the study mosquitoes, a plaque assay was performed. The following are supporting documents on the same.

**CHIKV stock titer**

**
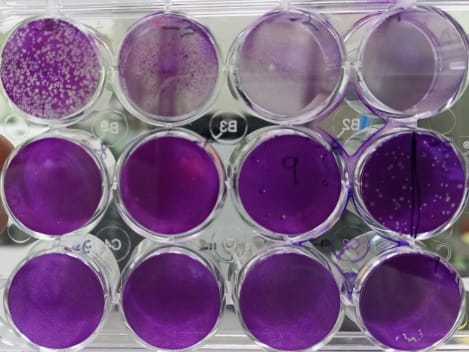
**

**Fig 1**. Plaque assay plate used to determine the titer of the CHIKV stock used to challenge *Ae. aegypti* mosquitoes from Kisumu and Busia Counties. The viral titer was converted to logarithm (10^6.95^ PFU/mL).


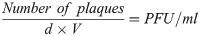
 (**Mulwa *et al* 2018)**

- No of plaques = 9
- Dilution factor =10^-5^
- Volume of virus diluted = 100ul (in ml) = 10^-1^

9 = 9 × 10^6^ PFU/mL

10^-5^ × 10^-1^

Convert to log 6 log 9 = 10^6.95^ PFU/mL

**Chikungunya virus titers summery**

**Table 1. Results the study virus titration if stock, infectious blood meal and titration of infectious blood meal after feeding.**

| Virus | Stock Titer | TB | TA |
| --- | --- | --- | --- |
| CHIKV | Log 10^6.95^ | Log 10^6.79^ | Log 10^6.77^ |

TB: Time before, this is the viral titer of the infectious blood meal before exposing it to the mosquito populations.

TA: Time after, this is the viral titer of the infectious blood meal after 45 minutes of exposer
